# Mitigating Representation-Dependent Degradation in Acoustic Signal-Based Fault Diagnosis of BLDC Motors via Classifier-Independent Waveform Restoration

**DOI:** 10.3390/s26217090

**Published:** 2026-11-09

**Authors:** Seon Min Yoo, Hwi Gyo Lee, Wang Ke Hao, In Soo Lee

**Affiliations:** School of Electronic and Electrical Engineering, Kyungpook National University, Daegu 41566, Republic of Korea; seon01@knu.ac.kr (S.M.Y.); bi05179@knu.ac.kr (H.G.L.); ws20000726@knu.ac.kr (W.K.H.)

**Keywords:** Brushless DC motor, acoustic fault diagnosis, acoustic signal denoising, waveform restoration, Wave-U-Net, industrial noise, noise robustness

## Abstract

Industrial noise can degrade acoustic fault diagnosis differently across input representations. This study evaluated whether classifier-independent waveform restoration could reduce this disparity in BLDC motor diagnosis. A Wave-U-Net denoiser was trained without classifier losses or outputs and applied before generating time-domain waveform and STFT magnitude inputs for six CNN, LSTM, and CNN–LSTM classifiers, which remained unchanged. The dataset comprised 13,790 one-second acoustic segments recorded from a single BLDC motor under normal operating conditions and bearing, eccentricity, and electrical fault conditions. Industrial noise environments were simulated by mixing the recorded motor signals with external noise recordings from the MIMII dataset. At 0 dB, restoration increased mean classification accuracy from 69.27% to 97.37% and reduced the mean performance gap between the two input representations from 48.61 to 1.01 percentage points. Improvements were also observed at evaluated SNRs below the training range and under noise types excluded from denoiser training. Grad-CAM and feature-space analyses indicated that restoration shifted decision patterns and class-wise feature structures toward those observed without added industrial noise. These findings support waveform restoration as a common preprocessing approach for reducing representation-dependent degradation under the evaluated conditions.

## 1. Introduction

Brushless DC (BLDC) motors are widely used in unmanned aerial vehicles, robotics, electric propulsion systems, and industrial automation equipment owing to their high efficiency, precise controllability, long service life, and high power-to-weight ratio [1,2,3]. Because motors are key drive components in these systems, motor faults can lead to performance degradation, operational interruptions, and safety issues. Therefore, continuous condition monitoring and early fault detection are essential for maintaining system reliability and safety [4], and fault diagnosis methods that combine sensor signals with artificial intelligence-based classification models have been actively investigated [5,6,7,8]. Current, voltage, vibration, and acoustic signals are commonly used for this purpose. Current and voltage reflect the electrical state of the motor, whereas vibration signals capture mechanical abnormalities such as bearing faults and eccentricity [9,10]. However, current and voltage measurements require access to the drive circuitry, while vibration sensors require physical attachment to the motor housing or surrounding structures [11,12].

In contrast, acoustic sensors capture sound radiated from the motor in a non-contact manner, enabling condition monitoring during operation without major modifications to the existing system [13]. Low-cost MEMS microphones further enable simple measurement setups, providing a practical alternative to contact-based sensors. However, because acoustic signals propagate through air, they contain not only information related to the motor condition but also components originating from ambient noise. When industrial noise overlaps with fault-related acoustic components, diagnostic features can be masked or distorted [14,15]. Nevertheless, many studies on acoustic fault diagnosis have trained and evaluated models using data collected under controlled conditions with limited noise [16,17,18]. Even when high accuracy is achieved under such conditions, comparable performance cannot necessarily be expected in practical industrial environments where sounds from multiple machines and background noise coexist. Therefore, evaluating the applicability of acoustic fault diagnosis to real industrial environments requires not only assessing diagnostic accuracy under controlled conditions but also characterizing performance degradation under industrial noise and investigating methods to mitigate such degradation.

In acoustic fault diagnosis, the same signal can be provided to a classifier in different forms, such as a time-domain waveform or a frequency-domain representation, and the choice of representation can affect diagnostic performance. This choice becomes particularly important under noisy conditions, as previous studies have shown that sensitivity to noise can vary with the input representation [19]. Noise can therefore affect diagnostic performance differently across representations. Such representation-dependent degradation differs from a simple reduction in overall accuracy. When the extent of noise-induced degradation varies between representations, the vulnerability of a diagnostic system to noise becomes dependent on the representation used for diagnosis. This issue is particularly relevant in practical environments, where the noise level of an input signal is often unknown in advance and the relative robustness of different representations may therefore be difficult to determine beforehand. A noise-induced performance gap between representations consequently introduces an additional source of uncertainty in diagnostic system design and motivates investigation into whether this gap can be reduced without relying on a particular diagnostic representation.

Existing approaches for maintaining diagnostic performance under industrial noise have developed along several directions, including improving the noise robustness of individual classifiers, exploiting complementary information from different signal representations, and jointly optimizing denoising and diagnosis. Although these strategies differ in their implementation, they generally aim to maintain or improve diagnostic performance under noisy conditions. Zhou et al. [19] employed a CNN with input dropout and a wide convolution kernel for time-domain signals and constructed an FDFM using characteristic frequencies in the frequency domain. The diagnostic outputs of the two models were subsequently fused using Dempster–Shafer evidence theory. Their results showed that frequency-domain features were relatively less sensitive to severe noise than time-domain features, and this difference was exploited to improve diagnostic performance by combining complementary information from the two representations. Zhao et al. [20] proposed ETSDAE, which constructs stacked denoising autoencoders for time-domain and frequency-domain data and applies different targeted denoising strategies to each domain. The deep features learned from the two domains are then integrated for diagnosis, while a mutual-information-based constraint is used to strengthen the relationship between the learned features and class targets. Thus, the two representations are processed separately before their information is combined to improve noise robustness.

More recently, approaches that jointly optimize denoising and diagnosis have also been proposed. Xiao et al. [21] generated a single denoised waveform from a noisy signal and subsequently extracted time-domain and frequency-domain features from the restored signal for fusion through adaptive gating. However, the denoiser, feature extractor, and classifier are jointly optimized using a single objective function incorporating denoising, classification, and contrastive losses. In addition, the classifier feature representation is incorporated into the denoising objective. Consequently, the restoration process is coupled to the downstream diagnostic model. More broadly, denoising-based studies differ in the degree to which the restoration module is coupled to the classifier. In two-stage approaches, the signal is first restored or denoised features are learned, after which the classifier is trained separately [22,23]. In joint-learning approaches, restoration and classification are optimized simultaneously under a common objective [21,24]. However, most two-stage approaches evaluate the restored signal using only a single classifier, whereas in joint-learning approaches, the restoration process is optimized using an objective that includes classification loss and therefore becomes dependent on a particular classifier.

Deep learning-based denoising can also be categorized according to the signal representation in which restoration is performed, independently of how the denoiser and classifier are coupled time-frequency-domain approaches transform signals into two-dimensional representations, such as the short-time Fourier transform (STFT), continuous wavelet transform (CWT), or Mel spectrogram, and perform denoising in that domain. Most of these approaches use only magnitude information as the restoration target [25,26]. The restored representation can be used directly for diagnosis in the time-frequency domain without reconstructing the waveform [26]. When waveform reconstruction from magnitude information is required, an additional procedure, such as the Griffin–Lim algorithm, is needed to estimate phase information [27]. Phase-preserving waveform reconstruction is also possible, but it generally requires complex-valued architectures that jointly process the real and imaginary components of the STFT [28] or additional regularization to enforce phase consistency [29].

In contrast, waveform-domain approaches directly take a one-dimensional waveform as input and reconstruct a clean waveform. This avoids constraining the restoration target to a specific time-frequency representation while retaining phase information within the waveform itself. Wave-U-Net, for example, was originally proposed to reconstruct waveforms directly in the time domain, addressing limitations of spectrogram-based approaches associated with phase information and dependence on a fixed time-frequency transformation [30]. In speech enhancement, direct waveform reconstruction has likewise been reported to preserve phase information without requiring a separate phase-reconstruction procedure [31,32].

Previous studies have generally combined information from different signal representations to improve diagnostic performance. However, differences in noise-induced performance degradation among these representations have received limited attention as a distinct subject of analysis. Moreover, when waveform restoration has been employed, the restored signal has typically been evaluated using a single classifier or a restoration process coupled with a specific classifier. Based on these research gaps, we hypothesize that a single classifier-independent denoiser can improve noise robustness across different classifier architectures and reduce the noise-induced performance gap among input representations. To test this hypothesis, this study separates waveform restoration from the downstream classifier and applies a common restoration operation to the waveform before generating the diagnostic representations. The resulting time-domain and frequency-domain diagnostic paths are evaluated independently rather than fused. This design allows the representation-dependent degradation caused by industrial noise to be quantified directly and permits evaluation of whether common waveform restoration can reduce the resulting performance gap. Wave-U-Net is employed as the restoration architecture because it directly reconstructs the waveform in the time domain [30]. The contribution of this study does not lie in proposing a new denoising architecture, but rather in establishing a framework in which waveform restoration is applied as a classifier-independent common preprocessing step before different diagnostic representations are generated. This separation is also motivated by practical deployment considerations. In industrial monitoring, diagnostic models may use different input representations and architectures to analyze the same acoustic signals. The proposed approach allows a single waveform denoiser to serve as a common preprocessing module across these models without requiring model modification or retraining, whereas joint learning couples denoiser training to a specific classifier.

Specifically, four BLDC motor conditions are considered, and combinations of time-domain waveform and STFT magnitude inputs with different classifier architectures are used to quantify the noise-induced performance gap between representations and its change after restoration. Generalization is further evaluated at SNRs outside the denoiser training range and for industrial noise types not included in denoiser training. In addition, SI-SDR, Grad-CAM, and feature-space analyses are used to examine the relationship between waveform restoration and changes in diagnostic performance, internal decision patterns, and feature-space structure.

The main contributions of this study are summarized as follows.

- Representation-dependent degradation under industrial noise is quantified by measuring the difference in diagnostic performance between time-domain waveform and STFT magnitude inputs.
- A classifier-independent waveform restoration approach is introduced to reduce this performance gap using a single denoiser applied before input representation generation, without modifying or retraining the downstream classifiers.
- The approach is evaluated at both the signal and diagnostic levels across multiple classifiers, including SNRs and noise types excluded from denoiser training. Grad-CAM and feature-space analyses are also used to interpret the effects of waveform restoration.

The remainder of this paper is organized as follows. Section 2 describes the proposed methodology, including the Wave-U-Net-based denoiser and the fault classification models. Section 3 presents the experimental setup, including data acquisition, industrial noise construction, and the wavelet-based denoising baseline. Section 4 reports and discusses the results, including denoising performance, noise-induced representation-dependent degradation and its mitigation, comparison with the conventional baseline, and interpretation of the recovered diagnostic information. Section 5 concludes the paper.

## 2. Proposed Methodology

### 2.1. Overall Framework

**Figure 1:**
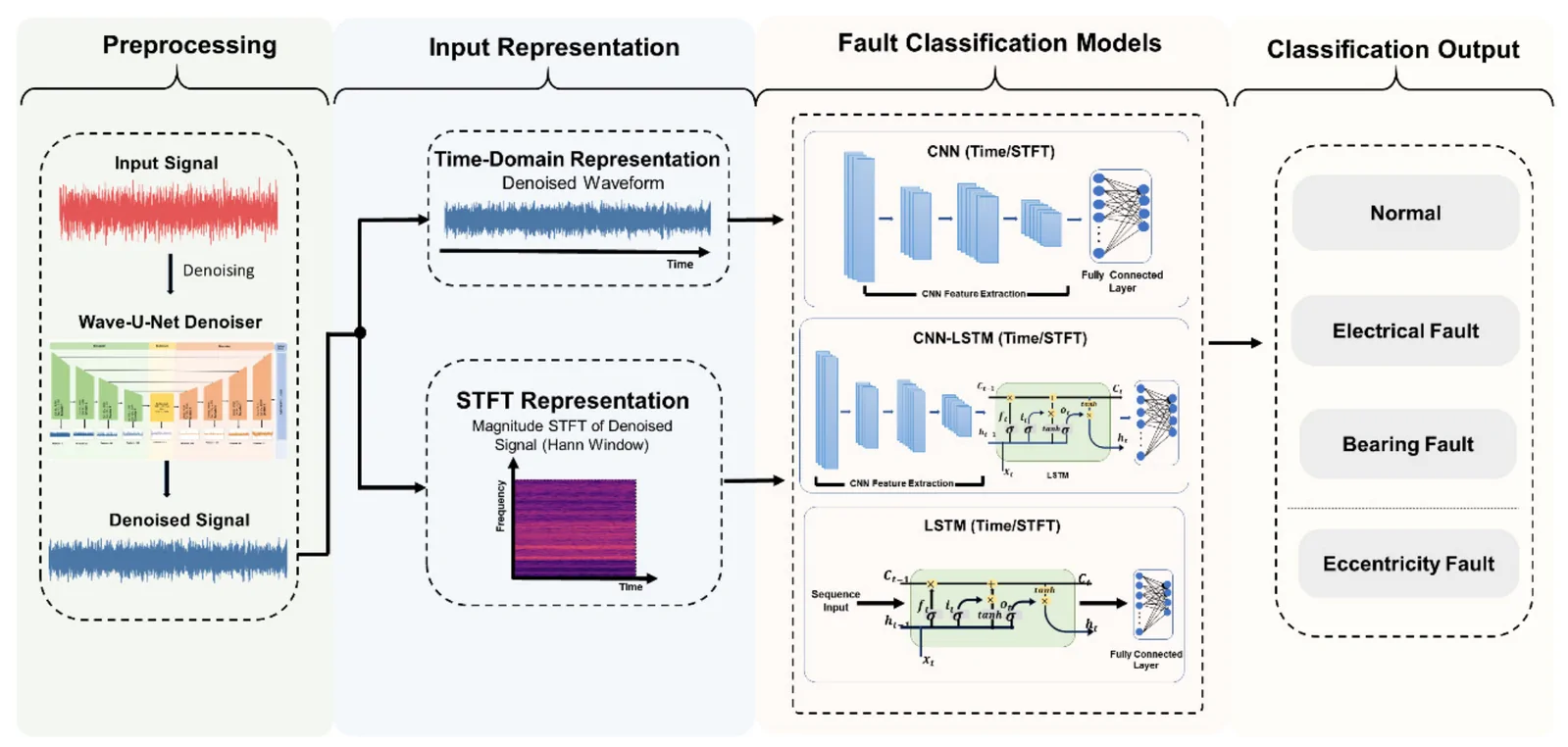
Overview of the proposed acoustic fault diagnosis approach. In the preprocessing stage, a common Wave-U-Net denoiser suppresses noise in the raw acoustic signal. In the input representation stage, the denoised signal is used either as a time-domain waveform or to generate an STFT magnitude spectrogram using a Hann window. Each representation is then independently fed into the CNN, CNN-LSTM, and LSTM classifiers to classify the motor condition as normal, electrical fault, bearing fault, or eccentricity fault. The shaded backgrounds distinguish the four processing stages. The arrows indicate the direction of signal flow, including the branching into time-domain and STFT representations.

The overall architecture of the proposed acoustic fault diagnosis system is illustrated in Figure 1. The system consists of four stages: signal preprocessing, input representation generation, fault classification, and classification output. The measured acoustic signal is first processed using a Wave-U-Net-based denoiser to restore the waveform in the time domain. The denoiser is applied uniformly as a preprocessing module, without first determining whether external noise is present, thereby producing a denoised waveform for subsequent classification.

A key feature of the proposed design is that waveform restoration is performed before the diagnostic input representations are generated. The restored waveform is used to construct two input representations. The time-domain waveform input is the output waveform of the Wave-U-Net, whereas the STFT magnitude input is obtained by applying a Hann-window-based STFT to the same denoised waveform and retaining its magnitude. Because both representations are derived from the same restored waveform, the effect of denoising can be evaluated under identical conditions for the two representations. This also enables analysis of how the performance gap between the representations changes in the presence of noise. Each representation is subsequently provided as input to three classifier architectures, CNN, LSTM, and CNN–LSTM, resulting in six classification models in total.

Each model classifies the input into one of four motor conditions: normal, electrical fault, bearing fault, or eccentricity. The denoiser and classifiers are trained independently. During denoiser training, noisy inputs are generated by mixing the original acoustic signals with external industrial noise, with the corresponding original signals serving as restoration targets. Neither classifier outputs nor classification losses are used to train the denoiser. The Wave-U-Net is therefore optimized exclusively for waveform restoration and remains independent of the downstream classifier architecture and diagnostic input representation. The CNN, LSTM, and CNN–LSTM classifiers are trained using only the original acoustic signals, without noise augmentation or denoising. Following training, a single fixed Wave-U-Net is used as a common preprocessing module for all six classification models; the classifiers are neither retrained nor fine-tuned using denoised inputs.

The effectiveness of the preprocessing is evaluated by comparing three input conditions—noiseless, noisy, and denoised—using the same fixed classifiers. The noiseless condition consists of the original recorded signals without added external noise. The noisy condition consists of the original signals mixed with external industrial noise, whereas the denoised condition consists of the noisy signals processed by the trained Wave-U-Net. This evaluation design enables separate assessment of the diagnostic performance degradation caused by external noise and the performance recovery achieved through denoising. It also enables a direct comparison of representation-dependent degradation and its mitigation between the time-domain waveform and STFT magnitude inputs.

### 2.2. Wave-U-Net-Based Denoiser

#### 2.2.1. Waveform Restoration Strategy

This study aims to analyze and mitigate the representation-dependent degradation caused by noise rather than to maximize the performance of a particular denoising method. To ensure a consistent basis for this analysis, the restoration applied before constructing the two diagnostic representations is kept identical. If restoration were performed separately for each representation, any change in the performance gap after restoration could reflect differences in the restoration processes rather than the effect of the representations themselves. Restoration is therefore performed directly on the waveform, which serves as the common source of both the time- and frequency-domain representations, before either representation is constructed. The same restored waveform can consequently be used to generate both representations, providing a controlled basis for comparing their diagnostic performance.

For this purpose, a one-dimensional Wave-U-Net-based denoiser is employed. Wave-U-Net performs waveform-to-waveform restoration using a multi-scale encoder–decoder architecture with skip connections. It captures long-range temporal correlations while preserving local waveform details, without first transforming the signal into a time-frequency representation [28]. These characteristics make it suitable for use as a common restoration stage before the two diagnostic representations are constructed. Accordingly, Wave-U-Net is used here as the waveform restoration module within the proposed classifier-independent preprocessing framework.

#### 2.2.2. Denoiser Architecture

**Figure 2:**
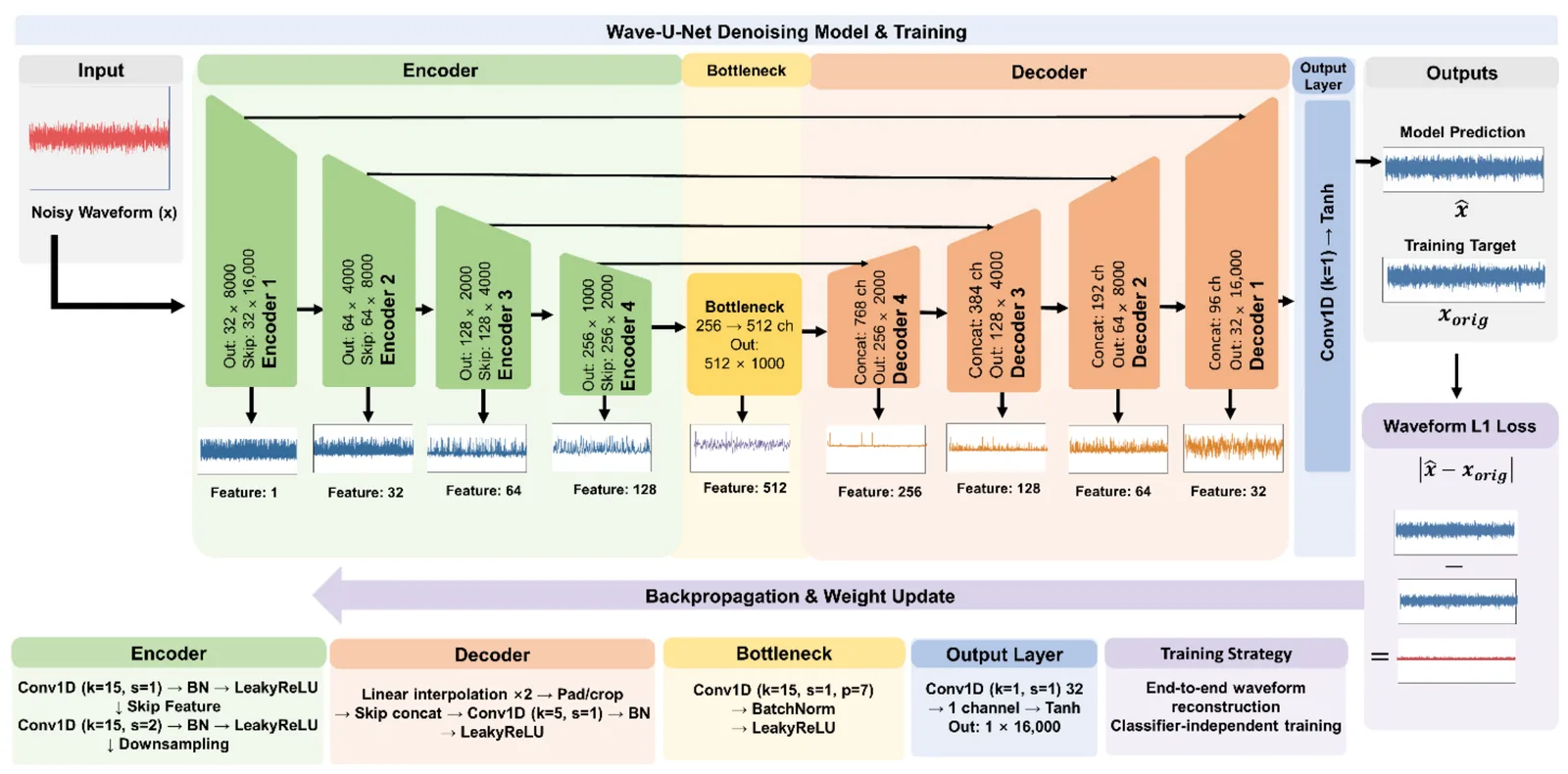
Overall architecture and training procedure of the proposed Wave-U-Net denoiser, comprising a four-stage encoder, a bottleneck, and a four-stage decoder with skip connections. The denoiser reconstructs clean waveforms by minimizing the waveform L1 loss without using classifier outputs or losses. Green, yellow, orange, blue, and purple indicate the encoder, bottleneck, decoder, output layer, and training-related components, respectively. Black arrows represent signal flow and skip connections, while the purple arrow indicates backpropagation and weight updates.

The overall architecture and training procedure are illustrated in Figure 2. The model receives a single-channel noisy waveform containing 16,000 samples and outputs a denoised waveform of the same length. The network comprises a four-stage encoder, a bottleneck, and a four-stage decoder. Skip connections link corresponding encoder and decoder stages to retain multiscale temporal features during waveform reconstruction.

The encoder progressively increases the number of feature channels from 32 to 256 (32, 64, 128, and 256), while reducing the temporal resolution. Each encoder stage comprises a stride-1 convolution for feature extraction followed by a stride-2 convolution for temporal downsampling. Both convolutions use a kernel size of 15 and are followed by batch normalization and LeakyReLU activation. The temporal length is therefore reduced successively from 16,000 to 1000 samples. The bottleneck then transforms the encoded representation into 512 feature channels while maintaining the temporal length at 1000 samples.

The decoder progressively restores the temporal resolution through linear interpolation with a scale factor of 2. At each stage, the upsampled feature map is concatenated with the corresponding encoder feature map through a skip connection and processed using a convolution with a kernel size of 5, followed by batch normalization and LeakyReLU activation. The number of output channels in the decoder decreases progressively from 256 to 32 (256, 128, 64, and 32). A final convolution with a kernel size of 1 reduces the feature representation to a single channel, followed by and a tanh activation that produces the denoised 16,000-sample waveform.

#### 2.2.3. Training Strategy

The denoiser was trained independently of the classifiers by minimizing the L1 loss between the reconstructed waveform and the corresponding noiseless target. Neither classifier outputs nor classification losses were incorporated into the training objective. This training strategy decouples waveform restoration from the downstream classification task, allowing a single trained denoiser to serve as a common preprocessing stage for classifiers using time- and frequency-domain inputs. The Wave-U-Net was trained using the Adam optimizer with an initial learning rate of 0.001 and a batch size of 64. A ReduceLROnPlateau scheduler halved the learning rate when the validation loss failed to improve for five consecutive epochs. Early stopping was triggered when the validation loss showed no improvement for 15 consecutive epochs.

#### 2.2.4. Denoising Objective Design

The denoiser training objective was designed based on the waveform L1 loss for direct waveform reconstruction. Several variants incorporating an additional spectral loss were also evaluated to impose an explicit constraint on frequency-domain reconstruction. The candidate objective functions are defined as follows.

Let *y* denote the noisy input signal and *x* its corresponding noiseless target signal. Let *f_θ_* denote the denoiser with parameters *θ*, and let $\hat{x}=\;f_{\theta }(y)$ denote the reconstructed waveform. The waveform L1 loss minimizes the sample-wise absolute difference between the noiseless and reconstructed waveforms:

(1) $$L_{\mathrm{wav}}=\frac{1}{T}\sum_{t=1}^{T}\left|{\hat{x}}_{t}-x_{t}\right|$$

where *T* denotes the total number of samples in the input signal. Because the waveform itself is used as the reconstruction target, the waveform L1 loss directly constrains the time-domain signal structure, including phase information.

Since the waveform L1 loss does not explicitly constrain frequency-domain reconstruction errors, an additional spectral loss was considered to promote spectral restoration. Multi-resolution STFT losses have been used to assess spectral reconstruction errors across different time–frequency resolutions [33]. In this study, the initial spectral objective combined linear- and log-magnitude L1 errors across multiple STFT resolutions. At the *k*-th STFT resolution, magnitude spectrograms of the noiseless and reconstructed signals are defined as

(2) $$M_{k}=\left|{\mathrm{STFT}}_{k}\left(x\right)\right|,\;{\hat{M}}_{k}=\left|{\mathrm{STFT}}_{k}\left(\hat{x}\right)\right|$$

The initial spectral loss was formulated as

(3) $$L_{\mathrm{spec}}^{\mathrm{init}}=\left(\frac{1}{K}\right)\sum_{k=1}^{K}\left[\left(\frac{1}{N_{k}}\right){\left\|{\hat{M}}_{k}-M_{k}\right\|}_{1}+\left(\frac{1}{N_{k}}\right){\left\|\log\left({\hat{M}}_{k}+\varepsilon \right)-\log\left(M_{k}+\varepsilon \right)\right\|}_{1}\right]$$

where *k* denotes the STFT resolution index, *K* is the total number of resolutions, *N_k_* denotes the number of elements at the *k*-th resolution, and *ε* is a small constant introduced for numerical stability in the logarithmic operation. Three FFT sizes, 512, 1024, and 2048 were used to obtain the multiple STFT resolutions. The initial training objective combining the waveform L1 and spectral losses was defined as

(4) $$L_{\mathrm{total}}^{\mathrm{init}}=L_{\mathrm{wav}}+\lambda_{\mathrm{spec}}L_{\mathrm{spec}}^{\mathrm{init}}$$

where λ_spec_ denotes the weighting coefficient for the spectral loss and was set to 0.01 or 0.03.

However, the absolute spectral errors in this formulation differ from the waveform L1 loss in both numerical scale and gradient magnitude and are also affected by the overall spectral magnitude. To reduce this dependence, a normalized multi-resolution STFT (MR-STFT) loss was further constructed by combining spectral convergence with a stabilized log-magnitude error [33]. At the *k*-th STFT resolution, the two terms are defined as

(5) $$L_{\mathrm{SC}}^{\left(k\right)}=\frac{{\left\|{\hat{M}}_{k}-M_{k}\right\|}_{F}}{{\left\|M_{k}\right\|}_{F}+\varepsilon }$$

and

(6) $$L_{\log}^{\left(k\right)}=\frac{1}{N_{k}}{\left\|\log\left(1+{\hat{M}}_{k}\right)-\log\left(1+M_{k}\right)\right\|}_{1}$$

where ‖⋅‖*_F_* denotes the Frobenius norm. Normalization by the target spectral norm reduces the dependence of the loss on the overall spectral magnitude. The final MR-STFT loss is obtained by averaging the two terms across the *K* STFT resolutions:

(7) $$L_{\mathrm{MRSTFT}}=\frac{1}{K}\sum_{k=1}^{K}\left[L_{\mathrm{SC}}^{\left(k\right)}+L_{\log}^{\left(k\right)}\right]$$

Because a fixed λ_spec_ does not directly control the relative contributions of the waveform and MR-STFT losses to parameter updates, a gradient-based balancing strategy was additionally considered. During an initial calibration stage, the gradient norms of the two losses were measured, and the spectral weight $\lambda_{\mathrm{spec}}^{*}$ was determined according to a predefined spectral-to-waveform gradient ratio *r*:

(8) $$G_{\mathrm{wav}}={\left\|\nabla_{\theta }L_{\mathrm{wav}}\right\|}_{2},\;G_{\mathrm{spec}}={\left\|\nabla_{\theta }L_{\mathrm{MRSTFT}}\right\|}_{2},\;\lambda_{\mathrm{spec}}^{*}=r\frac{G_{\mathrm{wav}}}{G_{\mathrm{spec}}}$$

Here, r represents the target ratio of the spectral-loss gradient contribution relative to that of the waveform loss, and $\lambda_{\mathrm{spec}}^{*}$ is the resulting spectral weight. The target ratios r = 0.10 and r = 0.20 were selected as two exploratory settings to introduce the spectral loss as an auxiliary constraint while retaining waveform reconstruction as the primary objective. At the initial calibration stage, these settings assigned the weighted spectral-loss gradient norms to 10% and 20% of the waveform-loss gradient norm, respectively.

During the initial calibration, *G_wav_* = 0.6364 and *G_spec_* = 5.4127 were measured, yielding $\lambda_{\mathrm{spec}}^{*}=0.01176$ and 0.02351 for r = 0.10 and r = 0.20, respectively. The gradient norms were measured only once, and the resulting spectral weight was then fixed throughout training. The corresponding MR-STFT-based objective is defined as

(9) $$L_{\mathrm{total}}^{\mathrm{MRSTFT}}=L_{\mathrm{wav}}+\lambda_{\mathrm{spec}}L_{\mathrm{MRSTFT}}$$

Unlike the initial spectral-loss formulation, in which λ_spec_ was directly specified, the MR-STFT formulation determines $\lambda_{\mathrm{spec}}^{*}$ from the target gradient ratio *r*. The candidate objectives were compared under the same fixed validation setting described in Section 4.1.1.

### 2.3. Fault Classification Framework

#### 2.3.1. Input Representation and Frequency-Band Selection

In this study, each 1-s acoustic signal was represented in two forms for classification: a time-domain waveform input and an STFT magnitude input. The time-domain waveform input consisted directly of the 16,000 amplitude samples acquired at 16 kHz, without additional feature extraction or transformation. For the STFT magnitude input, a Hann window was applied with an FFT size of 1024 samples and a hop length of 256 samples. Only the magnitude of the complex STFT coefficients was retained, with phase information excluded from the classifier input. The frequency range retained from the STFT magnitude spectrum was determined using the frequency-band selection procedure described below.

To identify candidate input frequency bands, the frequency-wise fault-class discriminability of the training data was analyzed. For each 1-s training segment, the STFT magnitude was averaged along the time axis to obtain one feature value for each frequency bin. The features from the 14 segments generated from the same original recording were then averaged to obtain a single recording-level spectral feature vector. Using these feature vectors, frequency-wise multi-class Fisher scores were calculated independently for the LOW, MID, and HIGH PWM conditions. The Fisher score at frequency *f* under PWM condition *p* was defined as the ratio of between-class variation to within-class variation [34] as follows.

(10) $$F_{p\left(f\right)}=\frac{\sum_{c=1}^{C}N_{c,p}{\left[\mu_{c,p}\left(f\right)-\mu_{p}\left(f\right)\right\}}^{2}}{\sum_{c=1}^{C}\sum_{i\in c}{\left[X_{\left(i,p\right)}\left(f\right)-\mu_{\left(c,p\right)}\left(f\right)\right]}^{2}+\varepsilon }$$

Here, *X*_(*i*,*p*)_(*f*) denotes the spectral feature at frequency *f* for recording *i* under PWM condition *p*, while *μ_c_*_,*p*_(*f*) and *μ_p_*(*f*) denote the mean of class *c* and the overall mean across all classes, respectively. *N_c_*_,*p*_ is the number of recordings belonging to class *c* under PWM condition *p*, *C* is the number of fault classes, and *ε* is a small positive constant introduced for numerical stability. To reduce scale differences among the Fisher scores obtained under different PWM conditions and limit the influence of extreme peaks before calculating common discriminability across conditions, the Fisher scores for each PWM condition were independently normalized by the 99th percentile of the corresponding distribution.

(11) $${\tilde{F}}_{p}\left(f\right)=\min\left(\frac{F_{p}\left(f\right)}{Q_{0.99}\left(F_{p}\right)},1\right)$$

To identify frequency regions with consistently high fault-class discriminability across all three PWM conditions, the geometric mean and minimum of the normalized Fisher scores were calculated.

(12) $$G\left(f\right)={\left[{\tilde{F}}_{L}\left(f\right){\tilde{F}}_{M}\left(f\right){\tilde{F}}_{H}\left(f\right)\right]}^{\frac{1}{3}}$$

(13) $$M\left(f\right)=\min\left[{\tilde{F}}_{L}\left(f\right),{\tilde{F}}_{M}\left(f\right),{\tilde{F}}_{H}\left(f\right)\right]$$

The geometric mean, *G*(*f*), reduces the influence of frequencies that exhibit high discriminability only under a specific PWM condition and reflects the balance of discriminability across the three conditions. In contrast, the minimum, *M*(*f*), takes the lowest score among the three PWM conditions and therefore represents the discriminability retained under the least favorable condition. Frequency regions with high values for both measures were consequently considered candidate regions containing consistent fault-class information across the PWM conditions.

Based on these candidate regions, four frequency bands with the same bandwidth of 2 kHz were constructed: 2–4, 3–5, 4–6, and 5–7 kHz. Because all four candidate bands had the same bandwidth, each STFT input consisted of 59 time frames and 129 frequency bins. The Fisher-based analysis was used only as a screening step to identify candidate frequency regions and was not used to directly determine the final frequency band. The final band was selected by comparing the noisy validation performance of STFT-based classifiers trained using each candidate band. Specifically, STFT-CNN, STFT-LSTM, and STFT-CNN-LSTM were independently trained for each frequency band using the original-signal training set and evaluated on the validation set with added fan noise at SNRs of 0, −2, −4, and −6 dB using five independent noise realizations. The candidate band with the highest mean validation accuracy across the three classifiers, four SNR conditions, and five noise realizations was selected as the STFT input band for the subsequent experiments. The test set was not used during frequency-band selection. The corresponding frequency-band selection results are presented in Section 4.3.

#### 2.3.2. Classifier Architectures

Six classification models were developed by combining CNN, LSTM, and CNN–LSTM architectures with time-domain and STFT inputs. Their configurations are summarized in Table 1.

All Conv1D layers used a stride of 1 and padding of (k − 1)/2 and were followed by batch normalization and ReLU activation. In both CNN–LSTM models, average pooling with a kernel size of 4 followed each of the first two convolutional blocks, while adaptive average pooling reduced the output of the third block to a length of 1. All nonadaptive pooling layers used strides equal to their respective kernel sizes. All LSTMs were unidirectional, and each classification head used ReLU activation between its two fully connected layers.

## 3. Experimental Setup and Evaluation Protocol

### 3.1. Acoustic Data Acquisition and Fault Implementation

**Figure 3:**
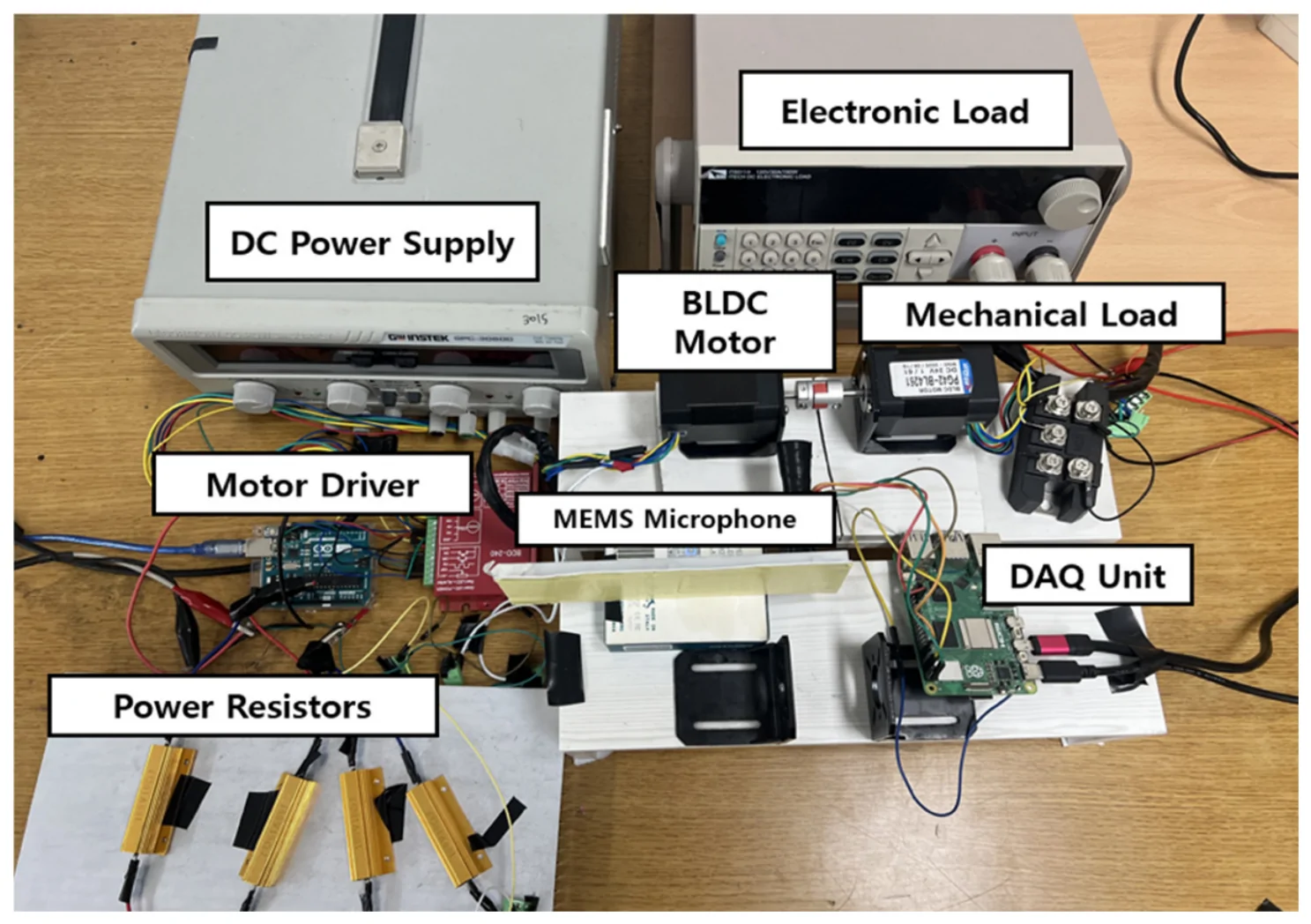
Experimental setup for BLDC motor acoustic signal acquisition.

Figure 3 illustrates the experimental setup used for acoustic data acquisition. A BL4261 three-phase BLDC motor was driven by a BDD-240 driver at 20 V. An identical BLDC motor mechanically coupled to the target motor was connected to an ITECH electronic load operated in constant-current mode at 0.1 A. Acoustic signals were acquired at a sampling frequency of 16 kHz using an INMP441 MEMS microphone positioned 4 cm from the side of the target motor and connected to a Raspberry Pi 5. The Raspberry Pi 5 was configured as a wireless access point, allowing recording initiation and file storage to be controlled remotely from a tablet through a custom web-based interface. Measurements were performed at low, medium, and high PWM driving levels. For each PWM level, the microphone position and orientation, load condition, and recording procedure were kept identical across all normal and fault conditions to ensure consistent measurement conditions.

Four motor conditions were investigated: normal operation, bearing fault, eccentricity fault, and electrical fault. The three fault conditions were implemented as follows.

Normal: The normal condition represents motor operation without any intentionally introduced fault.

**Figure 4:**
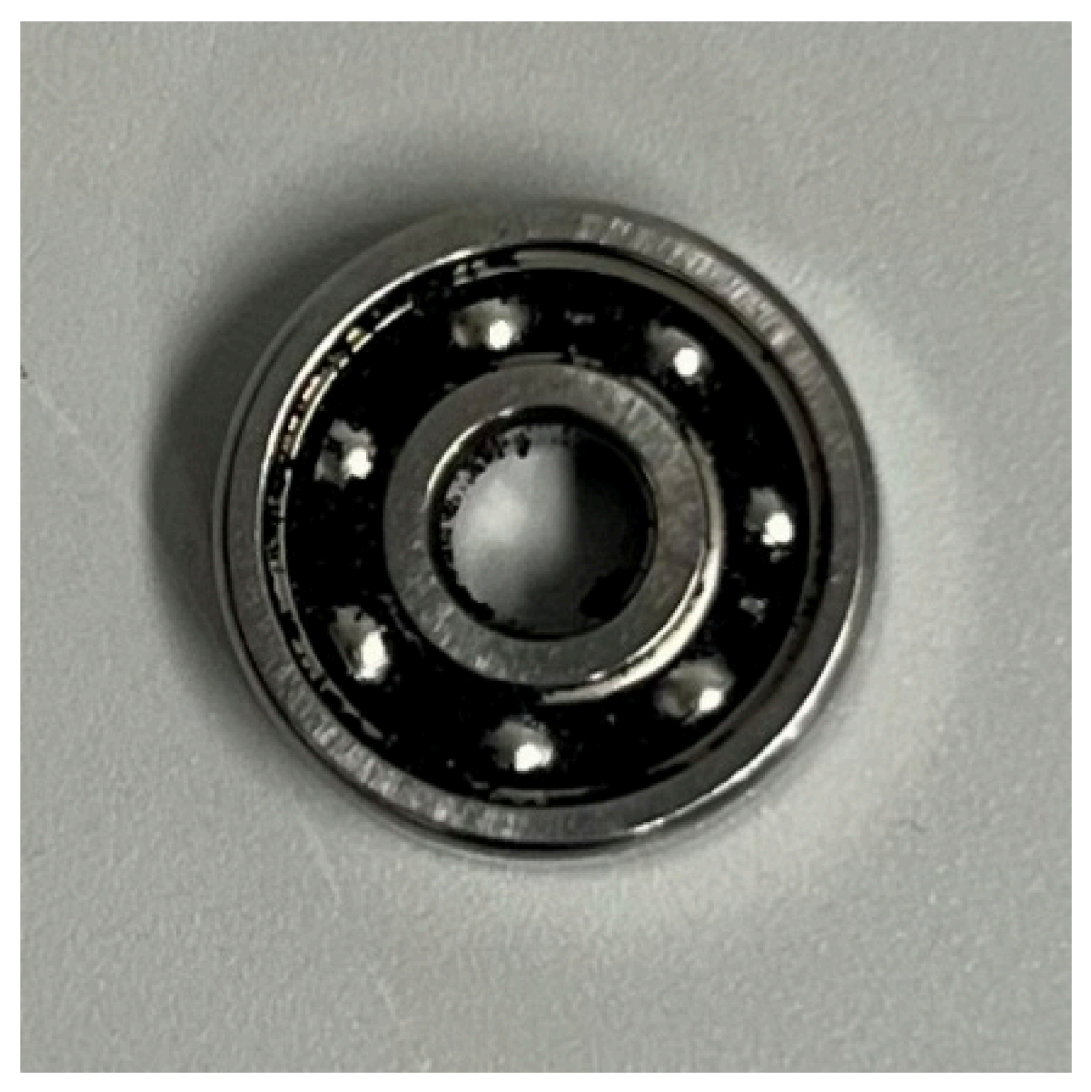
Bearing containing grease mixed with iron powder.

Bearing fault: Approximately 2.5 g of iron powder mixed with grease was injected into the load-side bearing to induce abrasive wear, as shown in Figure 4.

**Figure 5:**
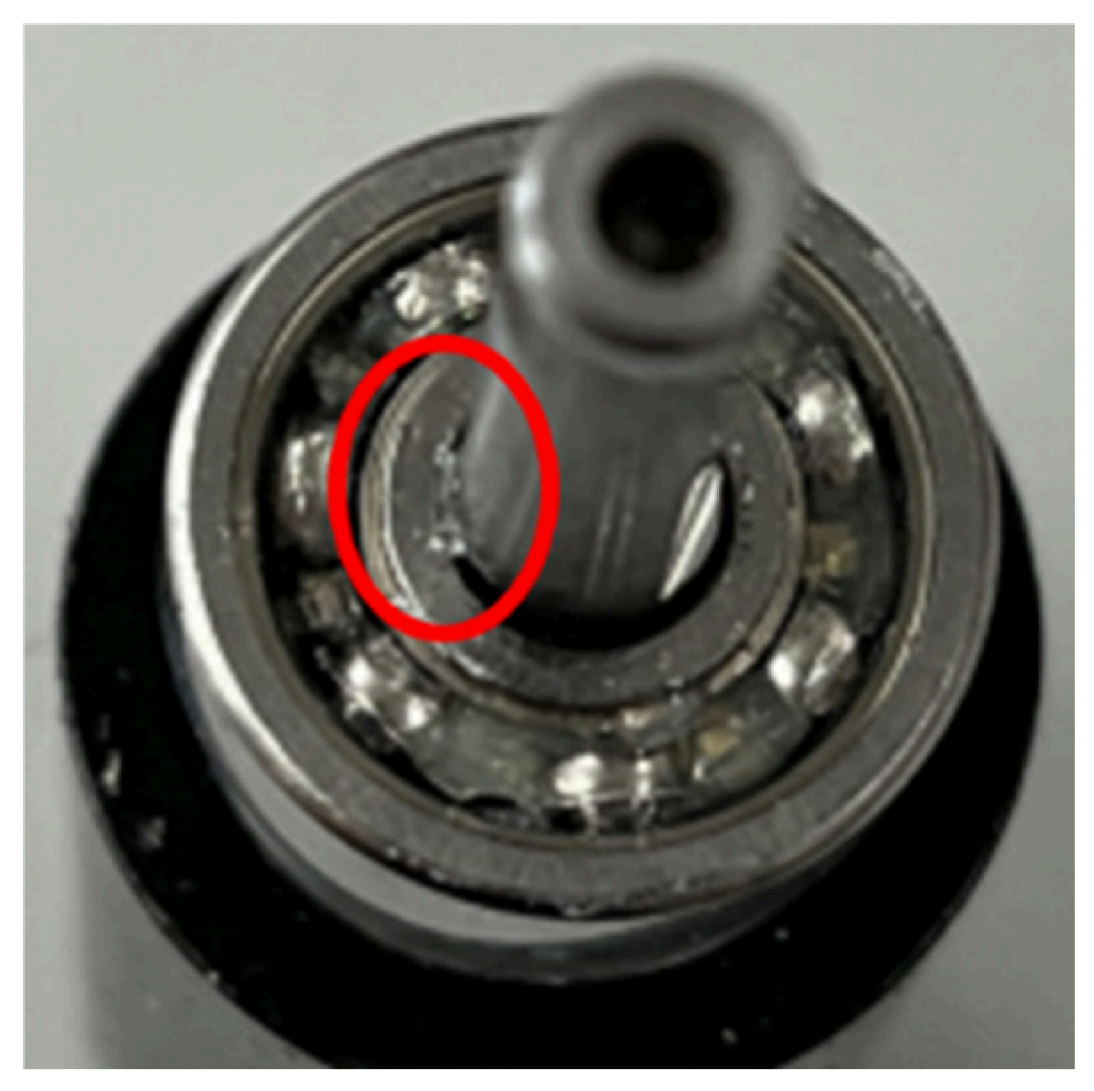
Eccentricity implementation by inserting a shim between the bearing inner race and the shaft. The red circle indicates the location of the inserted shim.

Eccentricity fault: After locally machining the rotor shaft surface, a thin metal shim was inserted between the shaft and inner race of the load-side bearing to induce dynamic eccentricity, as shown in Figure 5.

**Figure 6:**
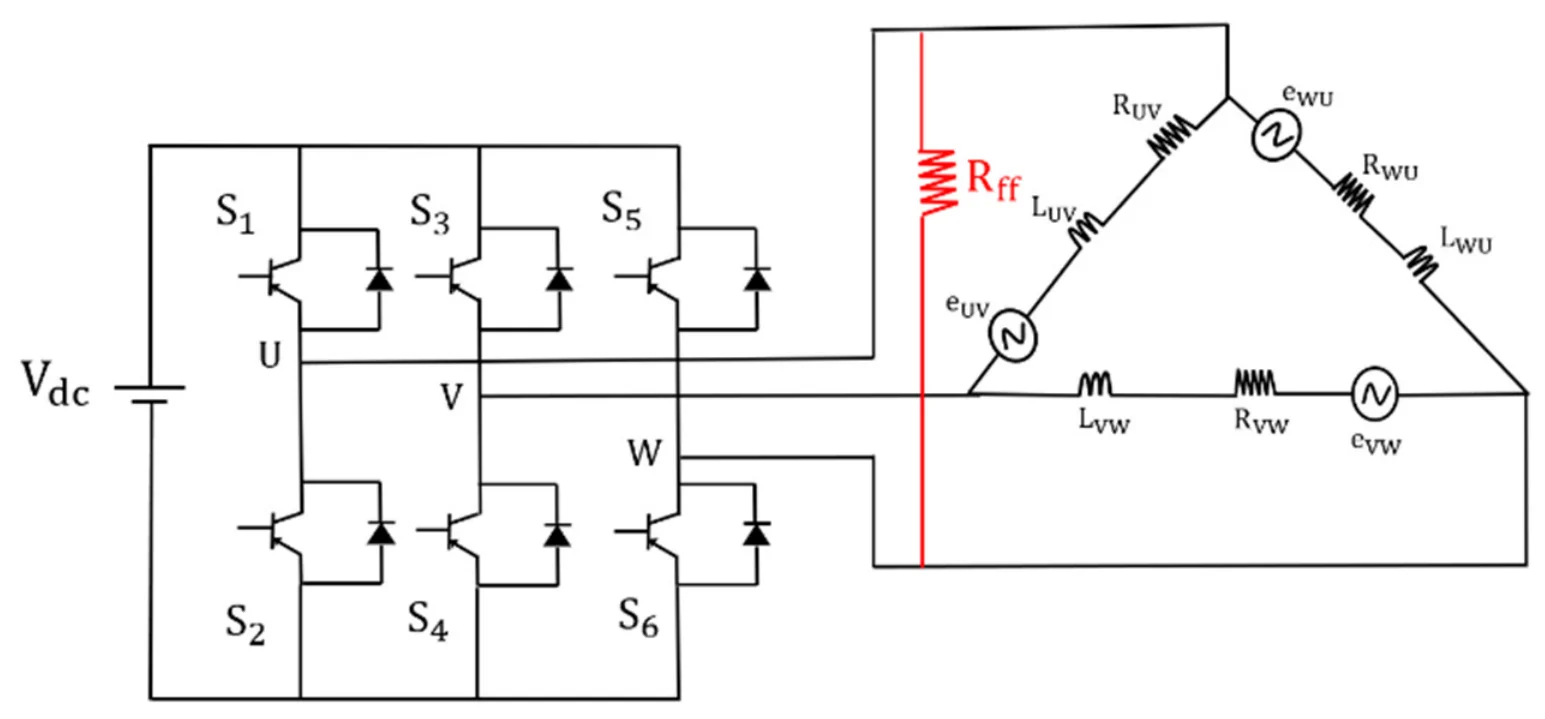
Electrical fault configuration with an external resistor *R_ff_* connected between the U and W phases of the delta-connected BLDC motor. The red branch highlights the fault-resistor connection. *V_dc_* denotes the DC supply voltage, *S*_1_–*S*_6_ represent the inverter switches, and U, V, and W identify the motor phases. *R*, *L*, and *e* represent winding resistance, inductance, and back electromotive force, respectively, with subscripts identifying the corresponding phase branches.

Electrical fault: Considering the delta-connected winding configuration, portions of the U- and W-phase wires near the motor terminal were exposed, and a 2.5 Ω external power resistor was connected between them to create an additional resistive current path. This configuration was used to reproducibly implement a U–W resistive interphase fault condition rather than directly induce an internal winding short circuit. The implemented configuration is shown in Figure 6. The acoustic measurements were intended to characterize motor operation under this externally imposed electrical condition. However, this configuration does not fully reproduce the localized electrical and thermal phenomena associated with an actual internal winding short circuit. Acoustic equivalence to an internal short-circuit fault was not directly evaluated, nor was the acoustic contribution of the resistor separately quantified. Therefore, the electrical-fault diagnosis results are interpreted within the scope of the implemented configuration.

### 3.2. Signal Segmentation and Dataset Partitioning

Each acoustic signal was recorded for 15 s. To exclude the initial transient period, the first 1 s of each recording was discarded, and the remaining 14 s were divided into 14 nonoverlapping 1-s segments. At a sampling frequency of 16 kHz, each segment contained 16,000 samples, resulting in a total of 13,790 acoustic segments.

The complete dataset was partitioned into training, validation, and test sets at ratios of 75%, 12.5%, and 12.5%, respectively. Stratified random splitting was performed based on the combination of fault class and PWM operating level (low, medium, and high) to maintain comparable distributions of motor conditions and operating levels across the three subsets. The partitioning was performed at the original-recording level, with all segments from each recording assigned to a single subset to prevent overlap of source recordings across the training, validation, and test sets. A fixed random seed was used to ensure reproducible partitioning. The validation set was used for early stopping and optimal model selection, whereas the test set was reserved exclusively for final evaluation of diagnostic performance. The same motor-data partition was used for the Wave-U-Net denoiser and all six classifiers. The denoiser was trained on noisy–clean pairs generated by mixing the training motor signals with fan noise, whereas the classifiers were trained independently on the same motor signals without added industrial noise. The motor test set remained fixed across all evaluated SNRs and noise types, and the trained denoiser and classifiers were applied without retraining or fine-tuning.

### 3.3. Industrial Noise Condition Construction

#### 3.3.1. External Noise Data and Dataset Separation

Industrial noise recordings were obtained from the MIMII dataset [35], comprising 1010 fan, 991 valve, and 1006 pump WAV files. The source dataset includes both stationary and non-stationary machine sounds. Channel 0 was extracted to obtain single-channel noise signals sampled at 16 kHz, matching the sampling rate of the original motor recordings. The fan-noise recordings were divided into mutually exclusive training, validation, and test subsets at the original WAV-file level. All noise segments extracted from a given file were restricted to its assigned subset, preventing segments from the same source recording from being shared across subsets. Valve and pump noise recordings were used exclusively for generalization testing and were excluded from denoiser training and validation, including model selection. To prevent data leakage between denoiser development and classifier evaluation, separate, nonoverlapping MIMII noise subsets were used for denoiser development and for generating noisy signals for classifier testing.

#### 3.3.2. SNR-Based Mixing

The noisy signal was generated by mixing the original motor signal with a scaled MIMII noise segment as follows:

*x_noisy_*(*t*) = *x_orig_*(*t*) + *α* × *n*(*t*) (14)

where *x_orig_*(*t*) denotes the original signal, *n*(*t*) denotes the MIMII noise segment, and *α* is the scaling factor. The SNR was defined as the ratio between the average power of the original signal to that of the scaled noise signal. Because the original and noise segments had the same length, *α* required to satisfy the target SNR was calculated directly from their *L*_2_ norms as follows:

(15) $$\alpha =\frac{{\left\|x_{\mathrm{orig}}\right\|}_{2}}{10^{\frac{{\mathrm{SNR}}_{\mathrm{target}}}{20}}{\left\|n\right\|}_{2}}$$

where ‖ · ‖_2_ denotes the *L*_2_ norm over each 1 s signal segment, and *SNR_target_* is the target SNR in decibels.

During denoiser training, 1-s MIMII noise segments were randomly selected, and the target SNR was randomly sampled from 0 to 15 dB. For validation, fixed noisy mixtures were generated at SNRs of 0, 3, 5, 8, 10, and 15 dB. For each original signal, the noise source and segment position were kept fixed across all candidate denoisers, with only the SNR varied. For classifier testing under fan noise, a separate fan-noise test subset was used to generate noisy signals at SNRs of 0, −2, −4, −6, −8, and −10 dB. For the unseen valve and pump noise recordings, the same SNR-based mixing procedure was applied to the fixed motor test set. Signal restoration was evaluated at −10, 0, and +10 dB, whereas classification performance was evaluated at 0 dB.

### 3.4. Wavelet-Based Denoising Baseline

To provide a conventional denoising baseline for comparison with the proposed Wave-U-Net, a discrete wavelet transform (DWT)-based denoising method using BayesShrink [36] was implemented. Three wavelet families, db4, db8, and sym8, were evaluated at decomposition levels of 4, 5, and 6. For each noisy signal, the noise standard deviation was estimated from the finest-scale detail coefficients using the median absolute deviation, and a BayesShrink threshold was independently determined for each detail subband. Soft thresholding was applied to the detail coefficients, while the approximation coefficients were retained without thresholding. Symmetric signal extension was used for both wavelet decomposition and reconstruction, with no additional normalization or amplitude clipping applied to the reconstructed waveform. The wavelet configuration was selected using only the validation data. The nine combinations of the three wavelet families and three decomposition levels were evaluated at SNRs of 0, 3, 5, 8, 10, and 15 dB using a fixed validation mixing procedure. The configuration with the highest mean SI-SDR improvement across these validation SNR conditions was selected. Neither the test data nor classifier performance was used during this selection procedure. Based on the validation results, sym8 with six-level decomposition, BayesShrink thresholding, and soft thresholding was fixed as the wavelet denoising baseline for subsequent comparison with the proposed method. The DWT–BayesShrink baseline does not involve iterative model-weight learning. Its wavelet and decomposition level were selected through validation, whereas the thresholds were estimated adaptively for each noisy input. The selected configuration was fixed before testing, and both denoising methods were evaluated using identical noisy test mixtures and the same pretrained classifiers without retraining. The comparison with the wavelet baseline is limited to the configurations evaluated in this study.

### 3.5. Training Configuration

All classifiers were trained exclusively on the original acoustic signals without added industrial noise or denoising. Cross-entropy loss and the Adam optimizer were used, with a batch size of 64, an initial learning rate of 0.001, and a maximum of 100 epochs. Dropout was applied immediately before each fully connected layer in all classification heads and, for the LSTM and CNN–LSTM models, between the two LSTM layers. The dropout probability was 0.3 for all models except the STFT-CNN–LSTM, which used 0.5. The learning rate was halved when the validation accuracy failed to improve for five consecutive epochs, and early stopping was applied when no improvement occurred for 15 consecutive epochs. For each classifier, the model with the highest validation accuracy was retained for evaluation. The architecture and hyperparameter settings were fixed for each model, with no model-specific hyperparameter search. These fixed classifiers were subsequently evaluated under the noisy and denoised conditions without retraining or fine-tuning.

## 4. Results and Discussion

### 4.1. Denoising Performance

#### 4.1.1. Selection of the Denoising Objective

The candidate objective functions defined in Section 2.2.4 were evaluated in two stages to determine the training objective used for the denoiser in all subsequent experiments. First, a preliminary screening was conducted during objective-function development to exclude configurations that showed clear degradation in restoration performance. Table 2 summarizes the progression of the objective-function design from waveform L1 loss to fixed-weight spectral objectives and normalized MR-STFT objectives with initial gradient-based weight calibration. It also presents the preliminary validation results and the candidates retained for the final comparison. Among the initial spectral-loss configurations, the larger fixed spectral weight of *λ_spec_* = 0.03 resulted in a substantial decrease in preliminary SI-SDRi and was therefore excluded from the subsequent controlled comparison. The waveform L1 baseline, the initial spectral objective with *λ_spec_* = 0.01, and the two redesigned MR-STFT objectives were retained.

Importantly, the preliminary validation setting used for Table 2 differed from the fixed validation mixtures used for the final comparison in Table 3. Therefore, the SI-SDRi values reported in the two tables are not directly comparable. After preliminary screening, all retained candidates were evaluated under an identical fixed validation setting to provide a controlled basis for final objective selection. Because the candidate objectives differed in formulation and numerical scale, the total validation loss was not used as a common selection criterion. Instead, all candidates were compared using the same signal-level restoration metrics computed on identical validation mixtures.

For the final comparison, 1,694 validation signals were evaluated under six SNR conditions of 0, 3, 5, 8, 10, and 15 dB. For each validation signal, the noise type and temporal segment used for noise addition were fixed, while only the SNR was varied. Waveform restoration performance was evaluated using waveform L1 error and scale-invariant signal-to-distortion ratio improvement (SI-SDRi). Frequency-domain restoration performance was assessed over the 0–8 kHz range using magnitude L1 error, log-magnitude L1 error, and spectral convergence. All frequency-domain metrics were computed using the same fixed STFT configuration for every candidate, with a Hann window, an FFT size of 4096, and a hop length of 512. This evaluation configuration was independent of the spectral formulations used in the candidate training objectives.

The final controlled comparison is presented in Table 3. Adding the initial spectral loss to the waveform L1 objective reduced SI-SDRi from 6.259 to 5.190 dB and increased the waveform L1 error from 0.03897 to 0.04366. The frequency-domain metrics also deteriorated, indicating that the additional spectral constraint did not improve reconstruction under the evaluated conditions. The redesigned normalized MR-STFT objectives substantially reduced this degradation, achieving SI-SDRi values of 6.225 and 6.236 dB for *r* = 0.10 and *r* = 0.20, respectively. However, neither configuration exceeded the waveform L1 baseline in terms of SI-SDRi or waveform L1 error. The MR-STFT objectives produced only marginal improvements in individual frequency-domain metrics, with the best values distributed across different configurations rather than showing consistent superiority over the waveform L1 baseline.

Overall, explicitly incorporating a spectral reconstruction constraint did not provide a consistent advantage over the waveform L1 alone. The waveform L1 objective achieved the highest SI-SDRi and the lowest waveform reconstruction error while maintaining frequency-domain reconstruction performance comparable to the more complex spectral objectives. Because the denoiser was intended to provide a common restored waveform for both diagnostic representations, higher SI-SDRi and lower waveform L1 error were prioritized over small improvements in individual spectral metrics. Accordingly, waveform L1 loss was selected as the training objective for all subsequent experiments.

#### 4.1.2. Signal-Level Restoration Performance

**Figure 7:**
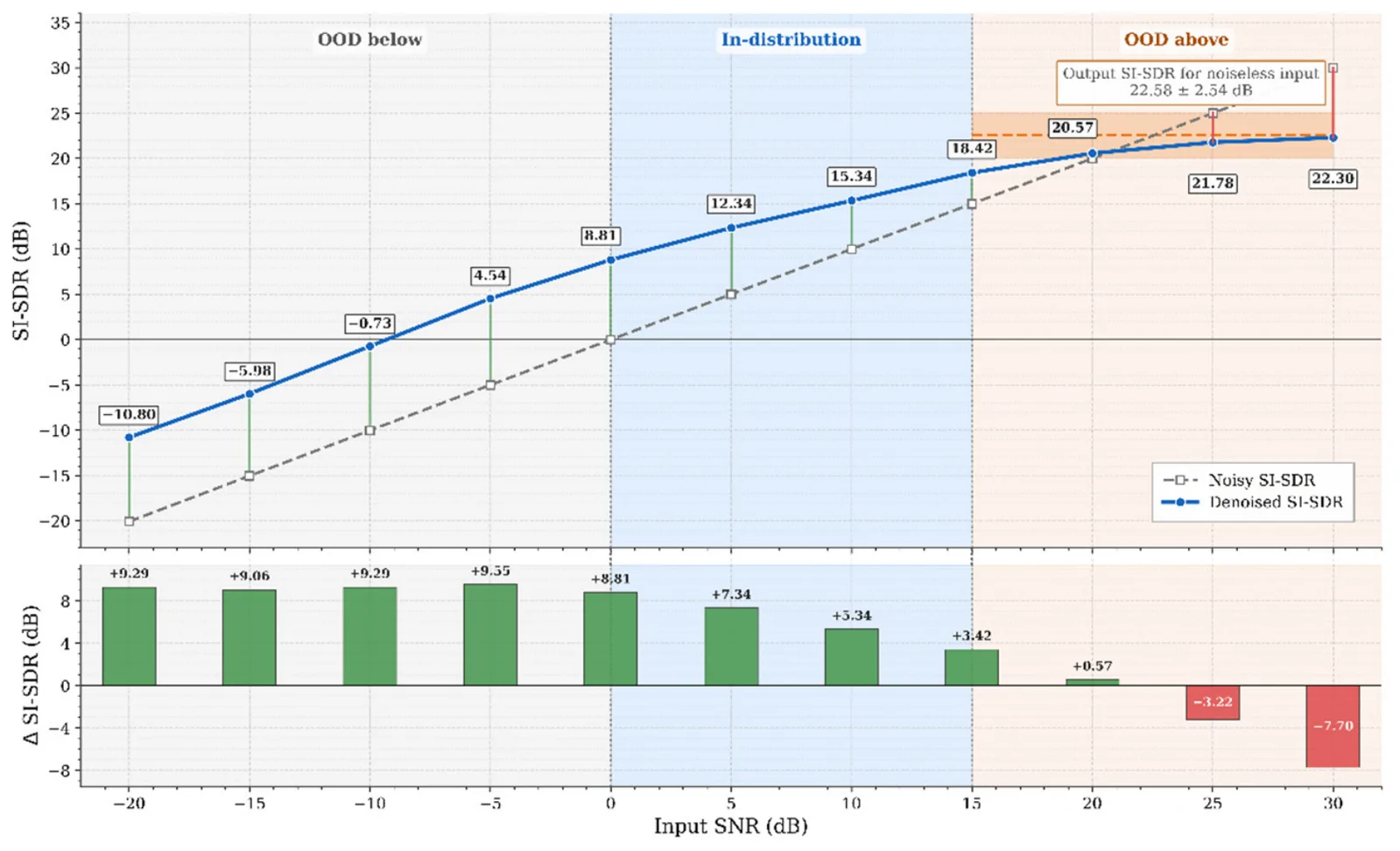
SI-SDR of noisy and denoised signals and the corresponding ΔSI-SDR across input SNR conditions from −20 to 30 dB. The shaded region indicates the 0–15 dB range used for denoiser training. The gray, blue, and orange shaded regions indicate input SNR ranges below, within (0–15 dB), and above the denoiser training range, respectively. The gray dashed and blue solid lines represent the SI-SDR of noisy and denoised signals, respectively, while the orange dashed line indicates the mean output SI-SDR for noiseless input. Green and red bars indicate positive and negative ΔSI-SDR values, respectively.

Before examining classification performance and the representation gap, we first assess whether the denoiser restores the signal at the waveform level, as this restoration provides the common basis for generating both diagnostic representations. To evaluate signal restoration independently of classification, SI-SDR was calculated for the noisy and denoised signals at each SNR using the corresponding noiseless signal as the reference. Because the denoiser was trained solely for waveform restoration, without classifier losses or output information, this analysis provides an assessment of signal-level restoration that is independent of any specific classifier architecture. The evaluation range was extended from −20 to 30 dB, encompassing the 0–15 dB range used for training, to examine the overall behavior of the denoiser both within and beyond its training range. The upper panel of Figure 7 shows the absolute SI-SDR values of the noisy and denoised signals, while the lower panel presents ΔSI-SDR, defined as the difference between the denoised and noisy SI-SDR.

Within the training range of 0–15 dB, indicated by the shaded region in Figure 7, the denoised SI-SDR exceeded the noisy SI-SDR at every evaluated SNR. ΔSI-SDR was +8.81 dB at 0 dB and +3.42 dB at 15 dB, indicating consistent improvement throughout the training range. Below the training range, from −20 to −5 dB, ΔSI-SDR remained between +9.06 and +9.55 dB. The denoiser therefore continued to provide substantial improvement outside its training range, without a sharp deterioration under more severe noise. However, the absolute denoised SI-SDR at −20 dB remained low at −10.80 dB. At such a low input SNR, strong noise may obscure motor-related waveform components, making accurate recovery difficult even when substantial noise reduction is achieved. Moreover, −20 dB is well outside the 0–15 dB training range, which may further limit restoration performance. Thus, performance at very low SNRs is better interpreted by considering both the magnitude of improvement and the absolute SI-SDR achieved after denoising. Above the training range, ΔSI-SDR progressively decreased as the input SNR increased. A positive improvement of +0.57 dB remained at 20 dB, whereas ΔSI-SDR became negative at 25 and 30 dB, reaching −3.22 and −7.70 dB, respectively. To examine this behavior, noiseless signals without added external noise were also passed through the denoiser. The resulting output SI-SDR was 22.58 ± 2.54 dB, indicating that the denoising process introduces some waveform distortion even when no external industrial noise is added. At high SNRs, the amount of removable noise decreases, while this processing-induced distortion becomes relatively more influential. Consequently, at 25 and 30 dB, the distortion outweighed the benefit of noise removal, resulting in lower SI-SDR for the denoised signals than for the noisy inputs. In this evaluation, 20 dB was the highest input SNR at which a positive improvement was observed, with ΔSI-SDR transitioning from positive to negative between 20 and 25 dB.

**Figure 8:**
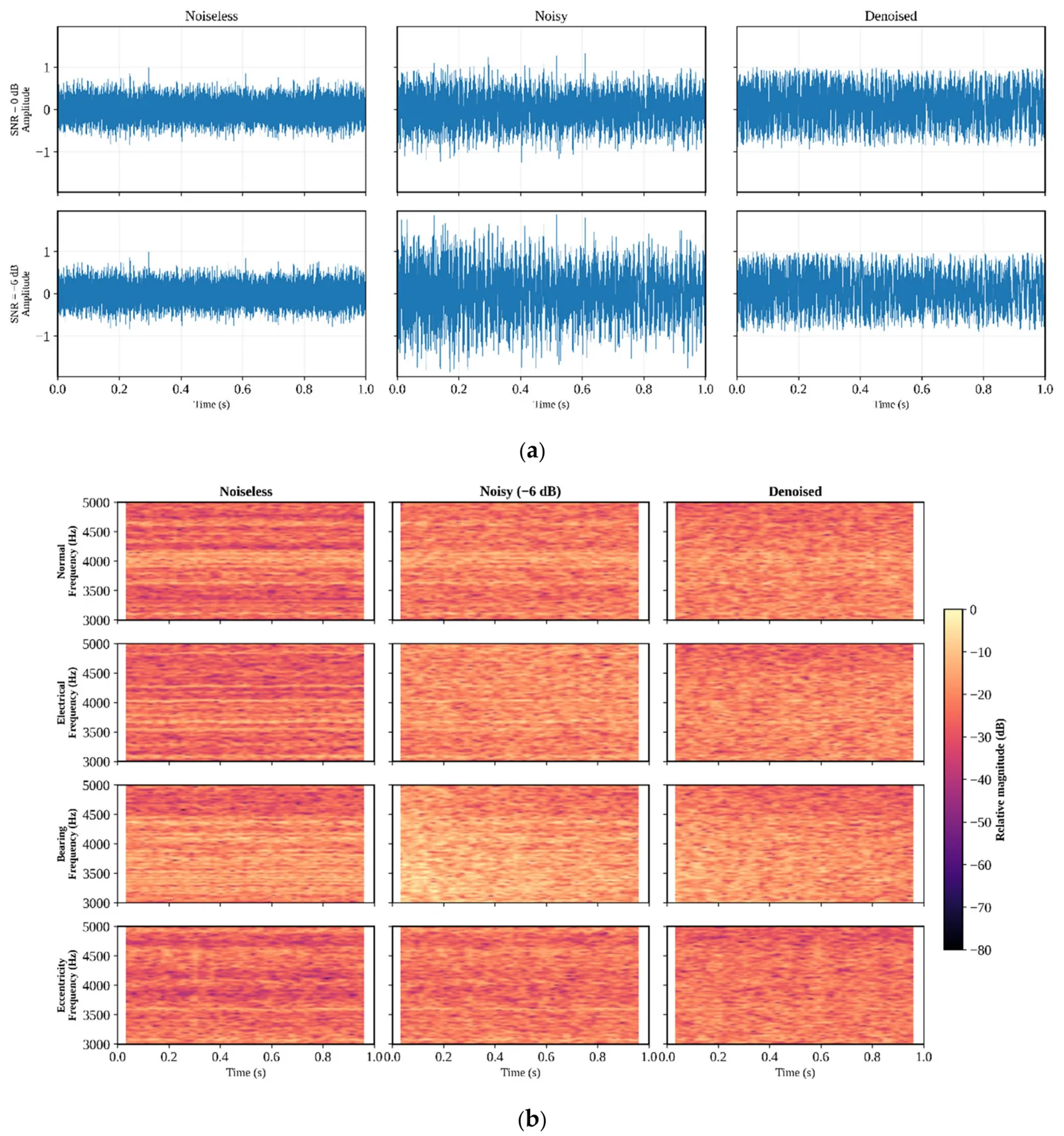
Signal-level comparison of the noiseless, noisy, and denoised conditions. (**a**) Time-domain waveforms of an example bearing-fault sample at 0 and −6 dB. The blue lines represent the acoustic waveforms under all three conditions. (**b**) 3–5 kHz spectrograms of the four fault classes (normal, electrical, bearing, and eccentricity) at −6 dB.

To visually examine how these restoration effects appear in the signals, Figure 8 compares the noiseless, noisy, and denoised conditions at the signal level. Figure 8a shows the time-domain waveforms of an example bearing-fault sample at 0 dB and −6 dB. Under the noisy condition, the amplitude fluctuations increased with decreasing SNR because of the added noise, with a more pronounced change at −6 dB than at 0 dB. After denoising, the excessive amplitude fluctuations were suppressed, and the overall waveform structure became closer to the noiseless waveform at both SNRs. Figure 8b compares the 3–5 kHz spectrograms, corresponding to the input band of the frequency-domain classifier, for the normal, electrical, bearing, and eccentricity classes at −6 dB. Under the noisy condition, the added industrial noise increased the spectral magnitude across the band, partially obscuring the class-dependent spectral structures observed under the noiseless condition. After denoising, these background spectral components were suppressed, and the spectral structures observed in the noiseless condition became more clearly visible across all four classes. Overall, these visual results show that waveform restoration applied before representation construction improves both the time-domain waveform structure and the frequency-domain structure within the diagnostic band, with both representations generated from the same restored waveform, consistent with the SI-SDR results presented above. However, signal-level restoration does not directly imply recovery of diagnostic performance. The following sections therefore examine the extent to which restoration recovers the classification performance of the time- and frequency-domain models and how this recovery affects the performance gap between the two representations.

To evaluate generalization to noise types not included in training, the fan-noise-trained denoiser was applied to valve and pump noises without retraining or adjustment and evaluated at −10, 0, and +10 dB, representing low, moderate, and high SNR conditions. As summarized in Table 4, both unseen noise types yielded positive ΔSI-SDR at all three SNR conditions, with minimum improvements of +4.26 and +3.63 dB for valve and pump noise, respectively. At 0 and +10 dB, the denoised signals achieved positive absolute SI-SDR values, reaching +14.25 and +13.63 dB for valve and pump noise, respectively. This indicates a substantial improvement in agreement with the noiseless reference even for noise types not included in training. In contrast, at −10 dB, although ΔSI-SDR remained positive, the absolute denoised SI-SDR remained negative at −1.40 and −3.50 dB for valve and pump noise, respectively. This behavior is consistent with the −0.73 dB obtained for fan noise at the same SNR. Across all SNR conditions, the improvement in SI-SDR decreased in the order of fan, valve, and pump noise, with the largest improvement observed for the fan noise used during training. Nevertheless, positive restoration effects were maintained for both unseen noise types. These results indicate that the denoiser retains its restoration capability for industrial noises not included in training, while also showing limited absolute restoration performance at −10 dB across all evaluated noise types.

### 4.2. Baseline Diagnostic Performance and Noise-Induced Degradation

**Figure 9:**
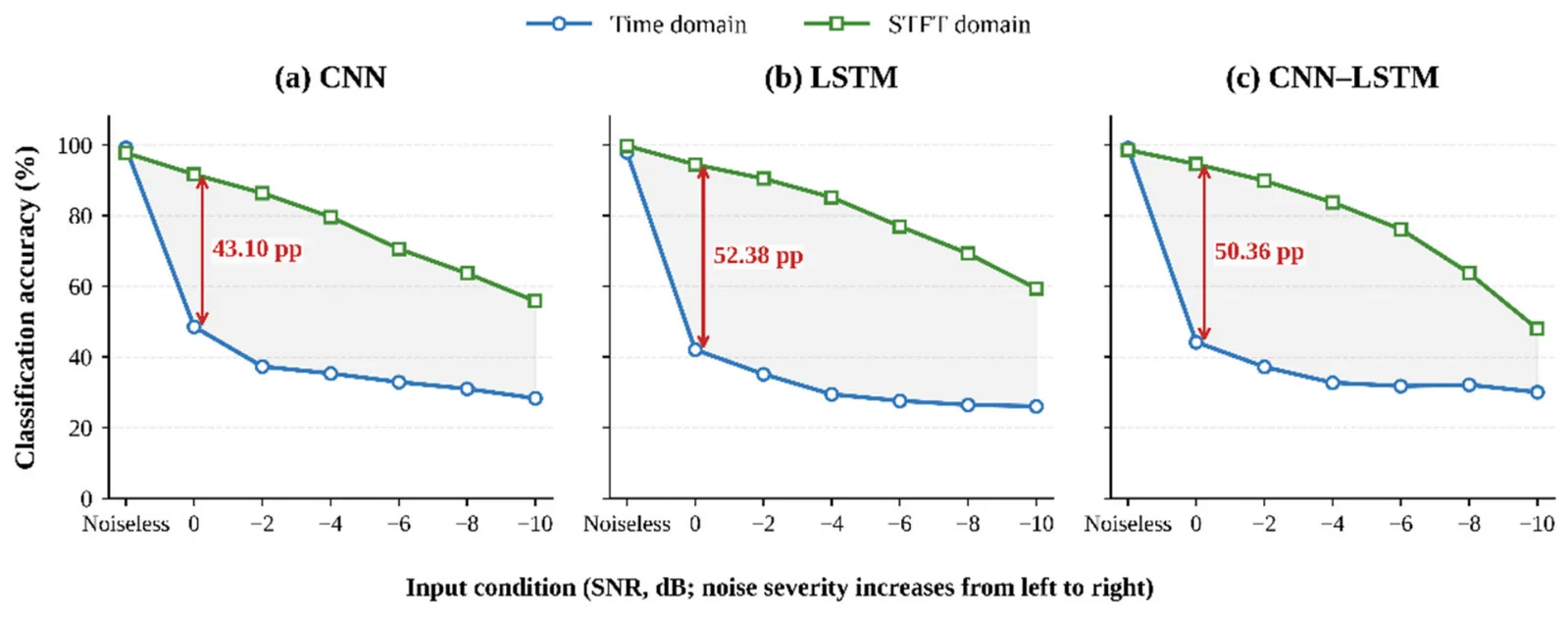
Classification accuracy degradation under noisy conditions across SNR for time-domain waveform and STFT magnitude inputs. (**a**) CNN, (**b**) LSTM, and (**c**) CNN–LSTM classifiers. The red double-headed arrows indicate the classification accuracy gaps between the two input representations at 0 dB SNR, expressed in percentage points (pp).

First, classification performance was evaluated under the noiseless condition, without added external industrial noise. As shown in Figure 9, the time-domain models achieved accuracies of 97.9–99.2%, while the frequency-domain models achieved 97.7–99.8%. Across the three classifier architectures, the differences between the two input representations remained within approximately 0.5–1.8 percentage points (pp). Thus, under the noiseless condition, both representations yielded consistently high diagnostic accuracy, with only limited differences in performance.

This close agreement was not maintained after external industrial noise was introduced. Figure 9 compares the accuracies of the time- and frequency-domain models across SNR conditions for the CNN, LSTM, and CNN–LSTM architectures. At 0 dB, the mean accuracy of the time-domain models decreased to 45.0%, representing a degradation of approximately 53.7 pp relative to the noiseless condition. In contrast, the mean accuracy of the frequency-domain models remained at 93.6%, with a decrease of only about 5.1 pp. Thus, under the same noise condition, the two input representations experienced markedly different levels of performance degradation, indicating representation-dependent degradation across all three classifier architectures.

The resulting performance gap between the two representations was also substantially larger at 0 dB. The accuracies of the frequency-domain models exceeded those of the time-domain models by 43.10, 52.38, and 50.36 pp for the CNN, LSTM, and CNN–LSTM architectures, respectively, giving an average gap of 48.61 pp. Thus, the relatively small performance gap observed under the noiseless condition increased substantially after the introduction of industrial noise.

As the SNR decreased further, the performance of the frequency-domain models also declined. At −10 dB, the mean accuracies of the time- and frequency-domain models decreased to 28.2% and 54.4%, respectively. Nevertheless, the frequency-domain models continued to achieve higher accuracy than the time-domain models, indicating that the performance gap between the two representations persisted even under severe noise conditions.

Overall, industrial noise reduced diagnostic performance and substantially increased the dependence of diagnostic accuracy on the input representation, as the two representations exhibited markedly different levels of performance degradation. Section 4.4 examines whether a common, classifier-independent waveform restoration stage can recover the noise-induced performance loss and reduce the performance gap between the time- and frequency-domain models.

### 4.3. Frequency-Band Selection Results

**Figure 10:**
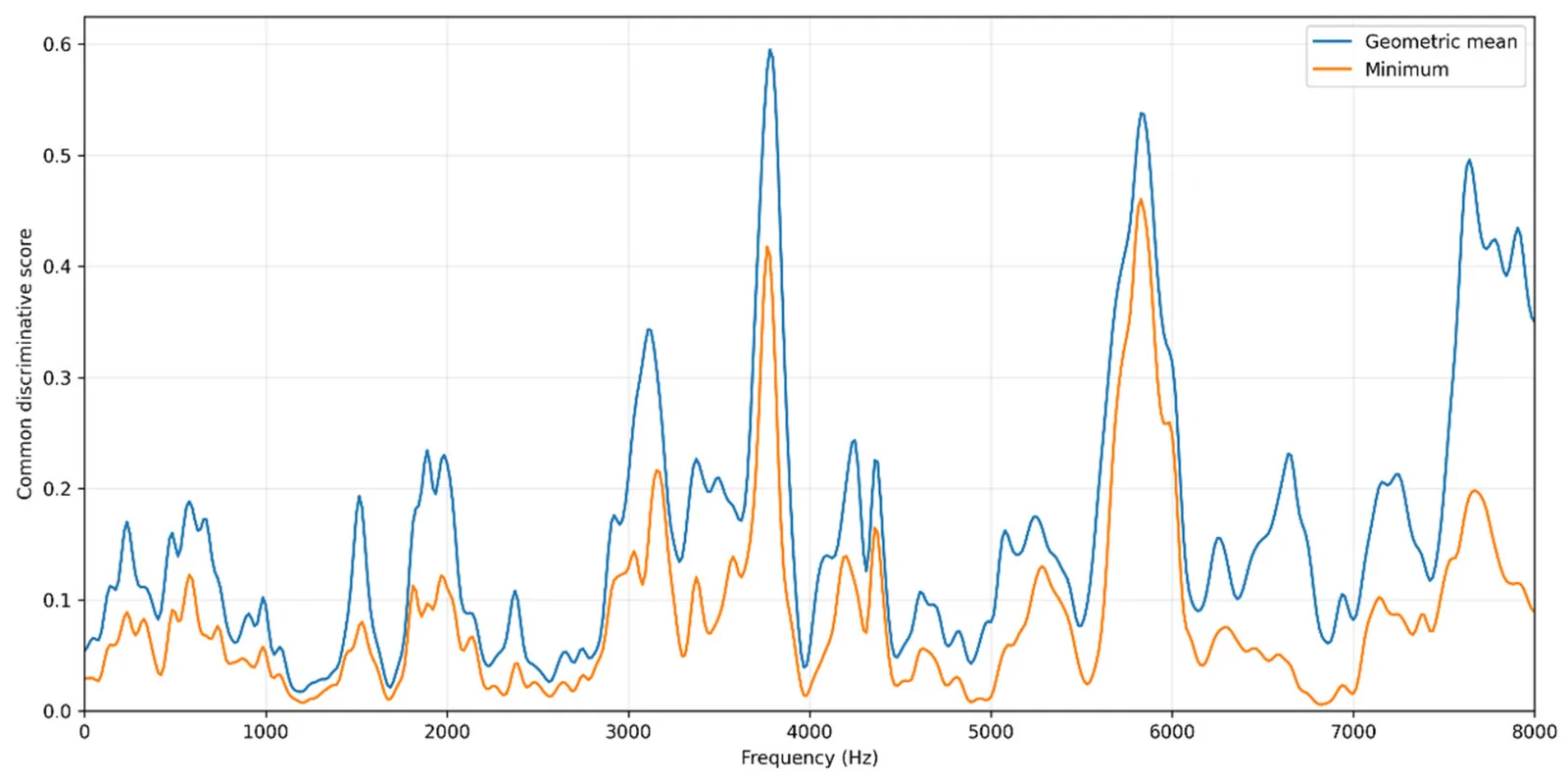
PWM-consistent frequency-wise fault-class discriminability calculated from the training recordings using the geometric mean and minimum of the normalized Fisher scores across the LOW, MID, and HIGH PWM conditions.

Figure 10 presents the frequency-wise fault-class discriminability across the PWM conditions, calculated from the training recordings using the geometric mean *G*(*f*) and minimum *M*(*f*) of the normalized Fisher scores. Both measures exhibited pronounced peaks around 3.8 and 5.8 kHz, indicating that these frequency regions provided consistently high class discriminability across the LOW, MID, and HIGH PWM conditions. Based on these results, the four candidate bands defined in Section 2.3.1, namely 2–4, 3–5, 4–6, and 5–7 kHz, were further evaluated using noisy validation performance.

However, the high Fisher-based discriminability observed in the noiseless training data did not directly translate into higher noisy validation accuracy. Table 5 summarizes the validation accuracy for each candidate band, averaged across the three STFT-based classifiers and five independent noise realizations at each SNR level. The 4–6 and 5–7 kHz bands, which include the discriminative region around 5.8 kHz, yielded overall mean validation accuracies of 68.606% and 60.864%, respectively. These values were lower than the 79.092% and 81.147% obtained for the 2–4 and 3–5 kHz bands, which include the region around 3.8 kHz. In particular, the 3–5 kHz band achieved the highest mean validation accuracy at each evaluated SNR of 0, −2, −4, and −6 dB, as well as the highest overall mean accuracy of 81.147%. Accordingly, the 3–5 kHz band was selected as the STFT input frequency range for all subsequent experiments.

### 4.4. Diagnostic Performance Recovery and Reduction in Representation Gap

**Figure 11:**
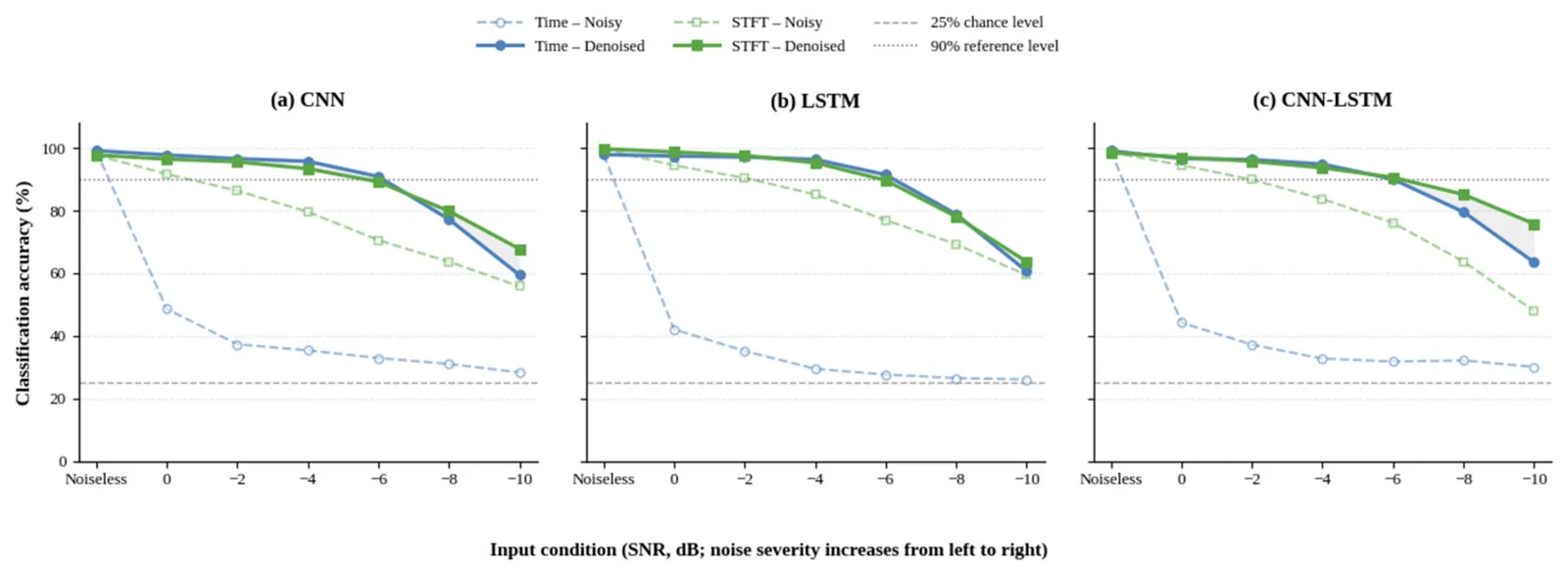
Classification accuracy recovery by denoising across SNR conditions according to input representation. (**a**) CNN, (**b**) LSTM, and (**c**) CNN–LSTM classifiers.

Figure 11 compares the classification accuracies under the noisy and denoised conditions across SNR levels for the CNN, LSTM, and CNN–LSTM architectures. The most direct effect of denoising was the reduction in the representation-dependent performance gap identified in Section 4.2. At 0 dB, the mean performance gap between the time- and frequency-domain models decreased from 48.61 pp under the noisy condition to 1.01 pp after denoising. For the CNN, LSTM, and CNN–LSTM architectures, the corresponding gaps decreased from 43.10, 52.38, and 50.36 pp to 1.31, 1.31, and 0.42 pp, respectively. As shown in Figure 11, the large separation between the time- and frequency-domain accuracy curves under the noisy condition was substantially reduced after denoising, with the effect being most pronounced at 0 dB. Thus, the noise-induced dependence on the input representation was largely reduced by a common waveform restoration stage without retraining or modifying the classifiers.

Importantly, the reduction in the performance gap was accompanied by recovery to high classification accuracy rather than convergence at a low performance level. After denoising, all six models achieved accuracies close to their respective noiseless levels at 0 dB, and this recovery was maintained down to approximately −6 dB, where the mean accuracy remained above the 90% reference level used for comparison. Below −6 dB, denoising continued to improve classification accuracy relative to the noisy condition; however, the mean accuracy progressively decreased and did not return to the noiseless level. Thus, at very low SNRs, waveform restoration was not sufficient to fully recover diagnostic performance, consistent with the limited signal restoration observed under extremely low-SNR conditions in Section 4.1.2.

To analyze the recovery of individual models in more detail, the recovery rate was defined as the proportion of the noise-induced accuracy loss recovered through denoising:

(16) $$\mathrm{Recovery}\mathrm{Rate}\left(\%\right)=\frac{{\mathrm{Acc}}_{\mathrm{denoised}}-{\mathrm{Acc}}_{\mathrm{noisy}}}{{\mathrm{Acc}}_{\mathrm{noiseless}}-{\mathrm{Acc}}_{\mathrm{noisy}}}\times 100$$

where *Acc_noiseless_*, *Acc_noisy_*, and *Acc_denoised_* denote the classification accuracies under the noiseless, noisy, and denoised conditions, respectively. A recovery rate of 100% indicates that the accuracy loss caused by noise is fully recovered to the noiseless performance level of the corresponding model.

**Figure 12:**
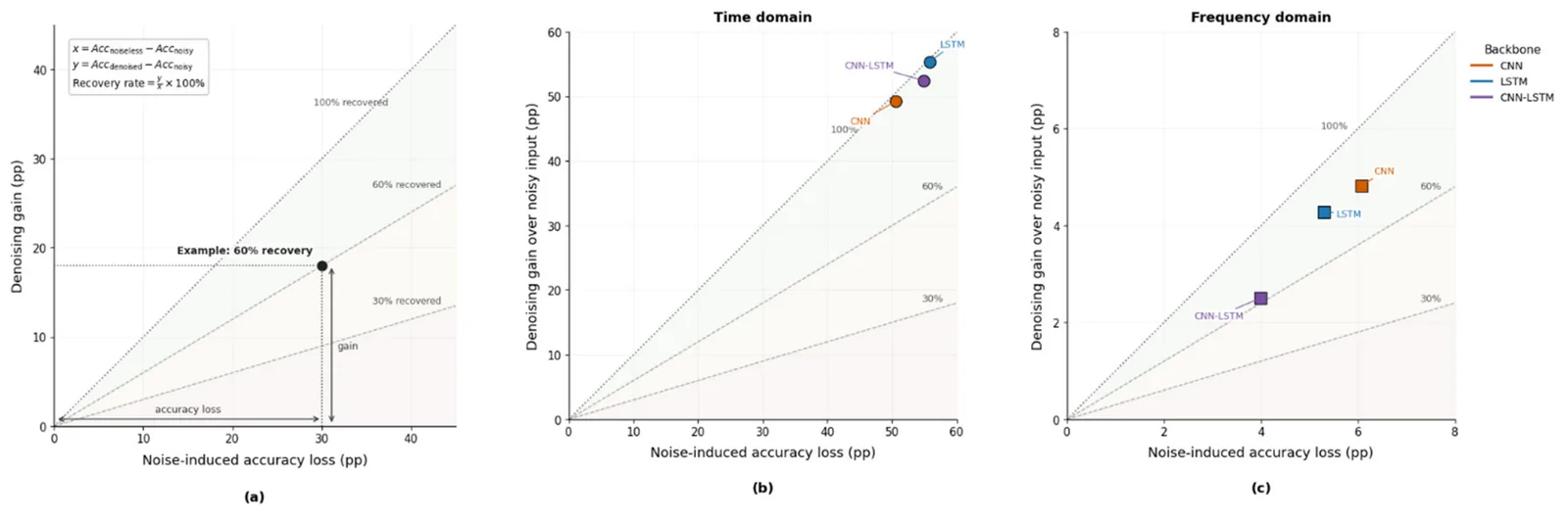
Accuracy loss–gain recovery maps at 0 dB SNR. (**a**) Definition of the recovery-rate coordinate system, where the horizontal and vertical arrows indicate the noise-induced accuracy loss and denoising gain, respectively. The diagonal reference lines represent different recovery rates, with the 100% line indicating full recovery, while the colored backgrounds distinguish recovery-rate regions. (**b**) Recovery map for the time-domain models. (**c**) Recovery map for the frequency-domain models.

Figure 12a defines the coordinate system used to calculate the recovery rate, while Figure 12b,c present the results for the time- and frequency-domain models, respectively. The horizontal axis represents the noise-induced accuracy loss, and the vertical axis represents the accuracy gain achieved through denoising. The diagonal line, *y* = *x*, corresponds to 100% recovery. For points below the diagonal, the vertical difference x − y represents the accuracy loss that remains unrecovered after denoising, expressed in percentage points. A smaller difference indicates that the denoised accuracy is closer to the corresponding noiseless baseline. Because the magnitude of the accuracy loss differed substantially between the two input representations, Figure 12b,c use different axis ranges to show their respective distributions clearly.

The two input representations exhibited different recovery patterns. The time-domain models incurred large accuracy losses of approximately 50 pp or more, yet all three models were located close to the 100% recovery line, indicating that most of the noise-induced loss was recovered. In contrast, the frequency-domain models incurred much smaller accuracy losses of approximately 4–6 pp, and all three models were located below the 100% recovery line, resulting in relatively lower recovery rates.

However, these lower recovery rates do not indicate lower final diagnostic performance. Because the noise-induced accuracy loss was small for the frequency-domain models, even a small residual difference between the noiseless and denoised accuracies had a relatively large effect on the recovery rate. The frequency-domain models nevertheless maintained high denoised accuracy despite their lower recovery rates relative to the time-domain models. The recovery rate is therefore a relative measure of the proportion of noise-induced accuracy loss recovered through denoising and is best considered together with the final denoised accuracy.

The noiseless, noisy, and denoised accuracies of the six models at 0 dB, together with the corresponding accuracy gains and recovery rates, are summarized in Table 6.

We further evaluated whether denoising extended the SNR range over which near-noiseless diagnostic performance could be achieved toward lower SNRs. For each model, performance within 1 pp of its corresponding noiseless accuracy was defined as the target criterion. The required input SNR was then estimated by linear interpolation between the two adjacent SNR conditions that bracketed this criterion. As summarized in Table 7, the average input SNR required to achieve near-noiseless performance decreased from 11.18 dB under the noisy condition to 0.75 dB after denoising, corresponding to an average reduction of 10.44 dB. Thus, denoising allowed near-noiseless diagnostic performance to be maintained at substantially lower input SNRs across all six models.

Overall, the common denoiser, which was trained independently of the classifiers, improved the classification accuracy of all six models across the evaluated SNR conditions, substantially reduced the noise-induced performance gap between the two input representations at 0 dB, and lowered the input SNR required to achieve near-noiseless performance by an average of 10.44 dB. These improvements were achieved without retraining or modifying the classifiers.

### 4.5. Comparison with a Wavelet-Based Denoising Baseline

To assess whether the diagnostic improvements obtained with Wave-U-Net could also be achieved using a conventional denoising method, the Wave-U-Net-based approach was compared with the wavelet-based baseline described in Section 3.4. Table 8 summarizes the SI-SDR and diagnostic accuracy results for the noisy, wavelet-denoised, and Wave-U-Net-denoised signals.

At the signal level, wavelet denoising did not improve SI-SDR under any of the evaluated SNR conditions. The mean SI-SDR decreased by 1.458 dB at 0 dB SNR and by 1.459 dB at −6 dB SNR, with SI-SDR improvements ranging from −1.616 to −1.419 dB across the evaluated conditions. In contrast, Wave-U-Net consistently improved SI-SDR, with improvements ranging from +8.811 to +9.559 dB. For example, at 0 dB, SI-SDR increased from −0.004 dB for the noisy signals to 8.807 dB after Wave-U-Net denoising, whereas wavelet denoising resulted in an SI-SDR of −1.462 dB.

A similar pattern was observed in diagnostic performance. The mean accuracy across the six classifiers decreased after wavelet denoising at every evaluated SNR level, with reductions of 6.895–20.863 percentage points relative to the noisy condition. In contrast, Wave-U-Net denoising increased the mean classification accuracy by 23.879–37.669 percentage points. At 0 dB, for example, the mean accuracy decreased from 69.266% to 48.403% after wavelet denoising, whereas Wave-U-Net denoising increased it to 97.371%. These comparisons show that adding a denoising stage did not consistently improve diagnostic performance. Under the evaluated conditions, the selected wavelet baseline reduced both SI-SDR and mean classification accuracy, whereas Wave-U-Net improved both measures. These results demonstrate the importance of evaluating preprocessing methods in terms of both waveform restoration quality and their effects on fixed downstream classifiers.

### 4.6. Robustness to Unseen Industrial Noise Types

The preceding analysis evaluated the recovery of diagnostic performance under fan noise, which was also the noise type used to train the denoiser. Although signal-level restoration under the unseen valve and pump noise conditions was confirmed in Section 4.1.2, a positive SI-SDR improvement alone does not necessarily translate into recovery of diagnostic performance. This section therefore examines whether the denoiser can also recover classification accuracy and reduce the performance gap between the input representations under these unseen noise conditions.

The input SNR was fixed at 0 dB, and the previously trained denoiser and the classifiers trained under the noiseless condition were applied without additional retraining. As shown in Table 9, the accuracies of all six models increased substantially after denoising for both unseen noise types. The average accuracy increased from 70.20% to 96.46% for valve noise and from 62.39% to 94.92% for pump noise, approaching the corresponding noiseless performance levels.

As observed for fan noise, the recovery in classification accuracy was accompanied by a reduction in the performance gap between the two input representations. Under the noisy condition, the average accuracies of the time- and frequency-domain models for valve noise were approximately 48% and 92%, respectively, indicating a large difference between the two representations. A similar pattern was observed for pump noise, for which the time-domain models experienced greater performance degradation than the frequency-domain models. After denoising, however, the accuracies of the two input representations became much closer, reaching approximately 95–97%, and the large performance gaps observed under the noisy condition were substantially reduced for both unseen noise types.

These results show that the recovery of classification accuracy and the reduction in the performance gap between the input representations were not limited to the fan noise used for denoiser training. When the denoiser trained only with fan noise was applied without retraining, it recovered the diagnostic accuracy of the existing classifiers and substantially reduced their dependence on the input representation under industrial noise types that were not included in training.

### 4.7. Interpretation of Diagnostic Information Recovery

#### 4.7.1. Grad-CAM Analysis of Decision Patterns

To investigate how industrial noise and denoising affect the importance patterns associated with classification decisions, Grad-CAM was applied to the last convolutional layer of the Time-CNN and STFT-CNN classifiers. For direct comparison across conditions, the Grad-CAM target class was fixed to the ground-truth class under the noiseless, noisy, and denoised conditions. Thus, each Grad-CAM profile represents the importance distribution associated with the same class score across all three conditions. For Time-CNN, Grad-CAM represents a temporal importance pattern over the waveform input. For STFT-CNN, it represents an importance pattern along the STFT time-frame direction for the 3–5 kHz STFT input representation.

To avoid selection bias that may arise from restricting the analysis to samples for which classification was recovered, the quantitative cosine-similarity analysis was performed over the entire set of 1680 test samples. Because cosine similarity is undefined for a zero vector, samples producing a zero Grad-CAM map under any of the three conditions were excluded only from the cosine-similarity analysis. This resulted in 1649 valid samples for Time-CNN and 1606 for STFT-CNN. The cosine similarities calculated using the corresponding noiseless Grad-CAM map as the reference are summarized in Table 10.

For Time-CNN, the cosine similarity to the noiseless reference increased from 0.593 ± 0.220 under the noisy condition to 0.933 ± 0.092 after denoising. This indicates that noise contamination substantially altered the temporal importance patterns associated with the ground-truth class, whereas after denoising these patterns became more similar to those observed under the noiseless condition. The change was particularly pronounced for the electrical, bearing, and eccentricity classes, whereas the normal class maintained relatively high similarity even under the noisy condition. Although similarity increased for all four classes, the extent of recovery varied across classes, with eccentricity showing a lower denoised similarity of 0.837 than the other classes.

**Figure 13:**
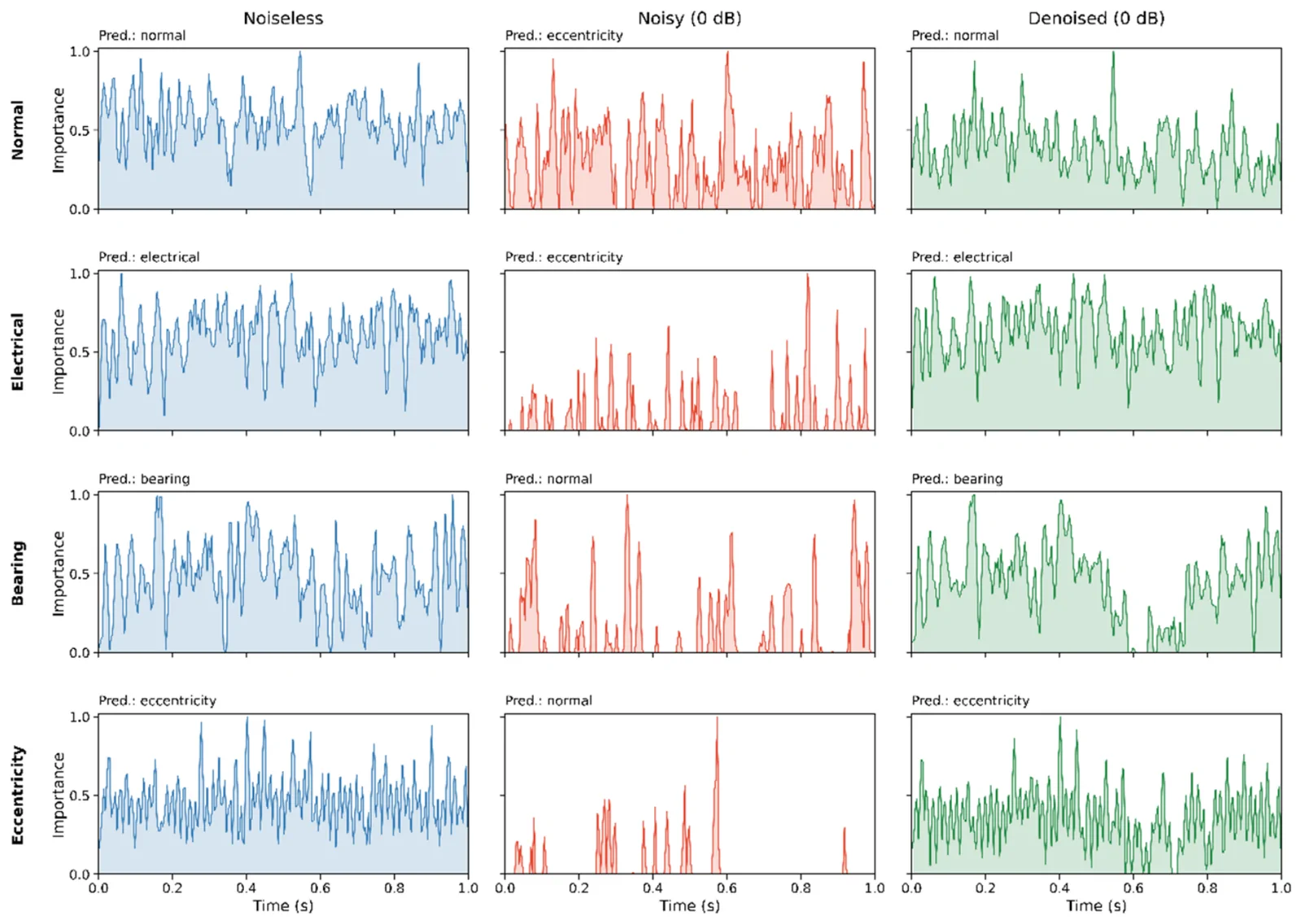
Grad-CAM temporal importance profiles of Time-CNN under noiseless, noisy, and denoised conditions at 0 dB. Class-wise median-recovery examples are shown using ground-truth-fixed Grad-CAM targets. Blue, red, and green indicate the noiseless, noisy, and denoised conditions, respectively.

Figure 13 presents selected Time-CNN classification-recovery examples for the four classes. Whereas the quantitative analysis was conducted over the full test set, Figure 13 provides a qualitative illustration of how classification recovery was accompanied by changes in the Grad-CAM patterns. To avoid dependence on extreme examples, the sample with the largest increase in cosine similarity was not selected. Instead, for each class, one classification-recovery sample closest to the median Grad-CAM cosine-similarity gain within that class was chosen.

Under the noiseless condition, each sample exhibited a temporal importance pattern associated with its ground-truth class. After contamination with 0 dB industrial noise, the corresponding importance pattern changed substantially, and the prediction shifted to an incorrect class. Following denoising, the importance pattern became more similar to its noiseless counterpart, accompanied by recovery of the correct prediction. These temporal patterns were interpreted only in terms of their distributional shape and similarity to the noiseless condition; no direct diagnostic meaning was assigned to specific temporal locations. For visualization, each Grad-CAM profile was independently normalized by its maximum value. In contrast, STFT-CNN maintained a high cosine similarity of 0.984 ± 0.070 under the noisy condition, which increased only slightly to 0.995 ± 0.031 after denoising. Thus, the Grad-CAM patterns of STFT-CNN remained highly similar to those under the noiseless condition even at 0 dB. This behavior is directionally consistent with the representation-dependent degradation observed in the classification results. For Time-CNN, the substantial accuracy degradation at 0 dB was accompanied by a marked decrease in Grad-CAM similarity, and both classification accuracy and Grad-CAM similarity increased after denoising. In contrast, STFT-CNN maintained both high classification accuracy and high Grad-CAM similarity under the noisy condition.

Overall, the recovery of Time-CNN classification accuracy after denoising was accompanied by a shift in the ground-truth-class-related temporal importance patterns toward those observed under the noiseless condition. The different Grad-CAM responses of the two models were also directionally consistent with the representation-dependent degradation identified in the preceding classification analysis.

#### 4.7.2. Degradation and Restoration of Class Structure in Feature Space

The Grad-CAM analysis showed that, after denoising, the internal decision-related patterns became more similar to those observed under the noiseless condition. To determine whether a similar change was also reflected in the learned feature space, features extracted immediately before the classification layer were compared under the noiseless, noisy, and denoised conditions. The Grad-CAM analysis showed that, after denoising, the internal decision-related patterns became more similar to those observed under the noiseless condition. To determine whether a similar change was also reflected in the learned feature space, features extracted immediately before the classification layer were compared under the noiseless, noisy, and denoised conditions.

**Figure 14:**
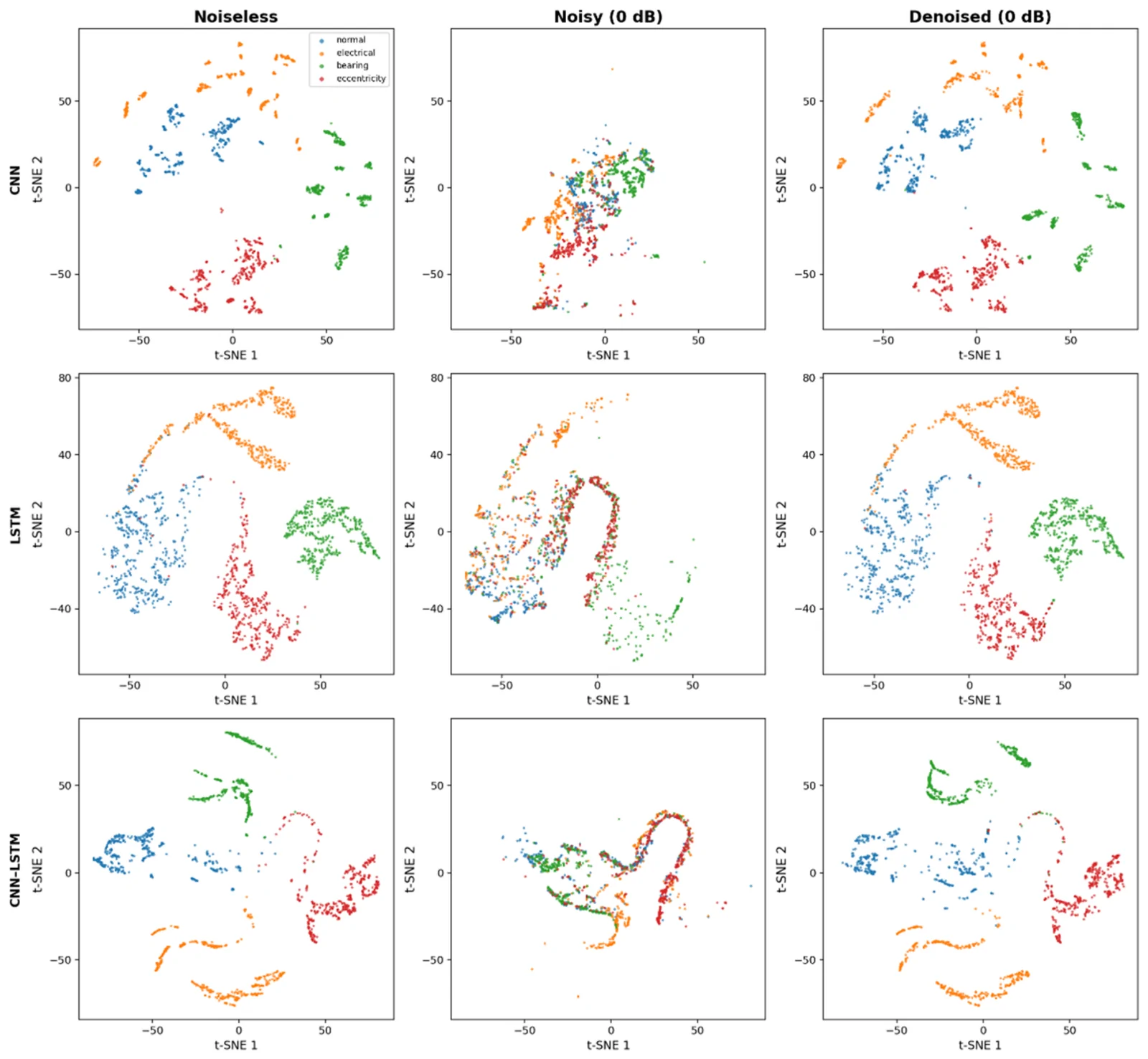
t-SNE visualizations of the feature spaces of the time-domain CNN, LSTM, and CNN-LSTM models under noiseless, noisy, and denoised conditions at 0 dB. For each model, features from the three conditions were jointly embedded into a common t-SNE space and displayed separately by condition.

Figure 14 presents t-SNE visualizations of the feature spaces of the three time-domain models: CNN, LSTM, and CNN-LSTM. For each model, features were extracted from all 1680 test samples under each of the three conditions. The features from the three conditions were jointly embedded into a common t-SNE space for each model and then visualized separately by condition. Because a two-dimensional t-SNE projection does not necessarily preserve class separability in the original high-dimensional feature space, these visualizations were used for qualitative comparison, while changes in feature-space structure across input representations and model architectures were evaluated quantitatively using silhouette scores computed from the original high-dimensional features (Table 11).

Under the noiseless condition, the three time-domain models formed clearly separated clusters corresponding to the four classes. Under the 0 dB noisy condition, these class-wise structures were substantially disrupted, resulting in increased overlap among the classes across all three models. After denoising, the clusters became more distinct, and the overall structures more closely resembled those observed under the corresponding noiseless conditions.

Because the two-dimensional t-SNE projection may not fully preserve class separability in the original high-dimensional feature space, class separability was quantitatively evaluated using silhouette scores calculated directly from the high-dimensional features before projection. The silhouette scores were computed using all 1680 test samples without sample filtering. Table 11 summarizes the silhouette scores of the six models under the three conditions. A higher silhouette score indicates greater separation between classes relative to within-class compactness.

The results reveal a clear representation-dependent difference in the extent of feature-structure degradation under noise. For the time-domain models, the silhouette scores under the 0 dB noisy condition decreased markedly from the noiseless values of 0.620, 0.852, and 0.891 to 0.064, −0.067, and 0.022 for CNN, LSTM, and CNN–LSTM, respectively, indicating substantial weakening of the class-separation structure. In particular, the negative silhouette score of Time-LSTM indicates that, on average, samples tended to be closer to the nearest cluster of another class than to their own class cluster. One possible explanation is that noise-induced perturbations in successive waveform frames affect the recurrent hidden-state updates of Time-LSTM, weakening class separability in the final time-step features used for classification. In contrast, local convolutions and average pooling in Time-CNN may attenuate some noise-induced variations, potentially explaining its better preservation of class structure under the evaluated condition.

In contrast, the silhouette scores of the frequency-domain models under the 0 dB noisy condition decreased to 0.561, 0.736, and 0.723 for CNN, LSTM, and CNN–LSTM, respectively. The corresponding decreases were 0.147, 0.161, and 0.184, substantially smaller than the decreases of 0.556, 0.919, and 0.869 observed for the time-domain models. Thus, although the STFT-based feature spaces were also affected by noise, the degradation of the class-separation structure was consistently smaller than that observed in the time-domain feature spaces across all three architectures. These results indicate that the learned feature spaces exhibited the same representation-dependent degradation pattern observed in classification accuracy.

After denoising, the silhouette scores recovered for all six models. The time-domain models recovered to 0.589, 0.841, and 0.859, approaching their corresponding noiseless values, while the frequency-domain models recovered to 0.659, 0.866, and 0.843. Thus, the recovery of classification accuracy after denoising was accompanied not only by changes in the final predictions but also by restoration of the class-separation structure within the internal feature spaces across both input representations and all three model architectures.

### 4.8. Computational Cost and Inference Time

The Wave-U-Net denoiser contains approximately 5.23 million trainable parameters and requires approximately 9.22 GMACs for its convolutional layers to process a 1-s acoustic signal sampled at 16 kHz, corresponding to 18.45 GFLOPs when one MAC is counted as two floating-point operations. Using FP32 precision and a batch size of one, the mean inference times were 255.19 ± 55.99 ms in the Google Colab CPU environment (using a single PyTorch thread, version 2.11.0) and 14.91 ± 0.18 ms on an NVIDIA Tesla T4 GPU. The corresponding real-time factors (RTFs), defined as the inference time divided by the input signal duration, were 0.2552 and 0.0149, respectively. Although waveform restoration introduces additional computational overhead, its mean processing time was shorter than the input signal duration in both environments. These measurements cover only the denoiser’s forward pass and do not represent the end-to-end latency of the fault diagnosis pipeline. Thus, the results support the computational feasibility of the restoration stage on the evaluated hardware, while real-time operation on resource-constrained edge devices requires further validation.

## 5. Conclusions

Representation-dependent performance degradation in acoustic fault diagnosis of BLDC motors under industrial noise was investigated, along with whether this degradation could be mitigated through classifier-independent waveform restoration. A Wave-U-Net denoiser trained solely for waveform restoration was applied as a common preprocessing module to six classifiers combining time-domain and STFT magnitude inputs with CNN, LSTM, and CNN–LSTM architectures, without retraining or modifying the classifiers. The two input representations exhibited markedly different levels of degradation under external noise, with the mean performance gap between the time- and frequency-domain models increasing to 48.61 pp at 0 dB. With the common denoiser, this gap decreased to 1.01 pp, mean accuracy across the six models increased from 69.27% to 97.37%, and the input SNR required for near-noiseless performance decreased by an average of 10.44 dB. These improvements were maintained outside the denoiser training range and under valve and pump noise excluded from denoiser training. In contrast, the conventional wavelet-based baseline improved neither signal restoration nor diagnostic accuracy and did not reproduce the gains achieved with Wave-U-Net. Grad-CAM and feature-space analyses further showed that, after restoration, diagnostic importance patterns and class-wise feature structures shifted toward those observed under noiseless conditions.

Overall, noise robustness in acoustic fault diagnosis depended substantially on the input representation and could not be fully characterized by overall accuracy alone. This representation dependence was reduced through classifier-independent waveform restoration without modifying the classifiers.

However, the SNR range over which signal restoration remained effective did not coincide with the range over which high diagnostic accuracy was maintained. High mean accuracy was maintained down to approximately −6 dB but declined at lower SNRs, indicating a limitation of waveform restoration alone under more severe noise conditions. The evaluation was limited to a single BLDC motor, four conditions, and three PWM operating levels, with only one baseline denoising method used for comparison. Future work could evaluate additional motors, speeds, and fault severities; investigate adaptive or selective denoising under more diverse industrial noise; and compare additional denoising methods under matched test conditions.

## Figures and Tables

**Table 1:** Classifier architectures for time-domain waveform and STFT magnitude inputs, where k denotes the convolutional kernel size.

Model	Input Representation	Input Organization/ Convolution Axis	Conv1D Channels	Kernel Sizes	Pooling	LSTM Layers/Hidden Size	FC Dimensions
CNN	Waveform	Full waveform; convolution along time	1 → 32 → 64 → 128 → 256	7/5/5/3	AvgPool(4) × 3; AdaptiveAvgPool(4)	—	1024 → 128 → 4
	STFT magnitude	Frequency bins as input channels; convolution along time	129 → 32 → 64 → 128 → 256	7/5/5/3	AvgPool(2) × 3; AdaptiveAvgPool(1)	—	256 → 64 → 4
LSTM	Waveform	Sequence of nonoverlapping 160-sample frames	—	—	—	2/128	128 → 64 → 4
	STFT magnitude	Sequence of STFT frames	—	—	—	2/128	128 → 64 → 4
CNN–LSTM	Waveform	1024-sample windows with a 512-sample hop; convolution along time within each window	1 → 32 → 64 → 128	7/5/3	AvgPool(4) × 2; AdaptiveAvgPool(1)	2/128	128 → 64 → 4
	STFT magnitude	Frame-wise spectra; convolution along frequency within each frame	1 → 32 → 64 → 128	7/5/3	AvgPool(4) × 2; AdaptiveAvgPool(1)	2/128	128 → 64 → 4

**Table 2:** Objective-function design stages and preliminary validation results.

Stage	Training Objective	Spectral Formulation	Balancing Strategy	r	Target $\left(\lambda_{\mathrm{spec}}^{*}\right)$	Warm-Up (Epochs)	Best Preliminary Val. SI-SDRi (dB) ↑	Screening Decision
Baseline	Waveform L1	–	–	–	–	–	6.289	Retained as reference
Initial spectral	L1+initial spectral loss	Linear-mag + log-mag	Fixed weight	–	0.01000	–	5.220	Retained
Initial spectral	L1+initial spectral loss	Linear-mag + log-mag	Fixed weight	–	0.03000	–	4.199	Excluded
Redesigned spectral	L1 + normalized MR-STFT	SC + stabilized log-mag	Gradient-based	0.1	0.01176	10	6.254	Retained
Redesigned spectral	L1 + normalized MR-STFT	SC + stabilized log-mag	Gradient-based	0.2	0.02351	10	6.266	Retained

Note: ↑ indicates that higher values are better; – indicates not applicable.

**Table 3:** Final comparison of restoration performance on identical fixed validation mixtures.

Training Objective	SI-SDRi (dB) ↑	Waveform L1 ↓	Mag. L1 ↓	Log-Mag L1 ↓	SC ↓
Waveform L1	6.259	0.03897	0.78886	0.34549	0.16863
L1 + original spectral loss (*λ_spec_* = 0.01)	5.190	0.04366	0.85911	0.35085	0.19192
L1 + normalized MR-STFT (*r* = 0.10)	6.225	0.03908	0.78793	0.34406	0.16823
L1 + normalized MR-STFT (*r* = 0.20)	6.236	0.03907	0.78937	0.34677	0.16740

Note: ↑ indicates that higher values are better; ↓ indicates that lower values are better.

**Table 4:** Denoised SI-SDR and SI-SDR improvement (ΔSI-SDR) for the training noise type (fan) and unseen noise types (valve and pump) under three input SNR conditions. Column SNRs denote the input mixing conditions. Denoised SI-SDR and ΔSI-SDR indicate restoration quality and improvement relative to the noisy input, respectively. The fan-noise results serve as a reference for the training noise type and were obtained using held-out recordings.

Noise Type	Denoised SI-SDR (dB)			ΔSI-SDR (dB)		
Input SNR	−10 dB	0 dB	+10 dB	−10 dB	0 dB	+10 dB
Fan (trained)	−0.73	+8.81	+15.54	+9.29	+8.81	+5.54
Valve (Unseen)	−1.40	+7.41	+14.25	+8.62	+7.41	+4.26
Pump (Unseen)	−3.50	+5.97	+13.63	+6.51	+5.97	+3.63

**Table 5:** Mean noisy validation accuracy of the candidate STFT frequency bands across three classifier architectures and five noise realizations.

Frequency Band (kHz)	Validation Accuracy (%)				Overall Mean (%)
	0 dB	−2 dB	−4 dB	−6 dB	
2–4	87.087	82.567	77.226	69.488	79.092
3–5	90.496	84.448	77.873	71.770	81.147
4–6	79.421	72.587	65.317	57.313	68.606
5–7	71.337	64.782	57.349	49.988	60.864

**Table 6:** Classification performance recovery at an input SNR of 0 dB.

Classifier	Input	Classification Accuracy (%)			Accuracy Gain (pp)	Recovery Rate (%)
		Noiseless	Noisy	Denoised		
CNN	Time	99.17	48.57	97.80	49.23	97.29
	STFT	97.74	91.67	96.49	4.82	79.41
LSTM	Time	97.92	42.08	97.44	55.36	99.15
	STFT	99.76	94.46	98.75	4.29	80.90
CNN-LSTM	Time	99.11	44.23	96.67	52.44	95.55
	STFT	98.57	94.58	97.08	2.50	62.69
Average		98.71	69.27	97.37	28.11	85.83

**Table 7:** Input SNR required to achieve classification accuracy within 1 percentage point of the corresponding noiseless performance.

Classifier	Input Representation	Input SNR Required for Near-NOISELESS Accuracy (dB)		Reduction in Required Input SNR (dB)
		Noisy	Denoised	
CNN	Time	15.79	0.83	14.96
	STFT	8.95	0.93	8.01
LSTM	Time	14.55	−2.58	17.13
	STFT	6.84	0.04	6.80
CNN-LSTM	Time	17.42	3.46	13.96
	STFT	3.57	1.82	1.75
Average		11.18	0.75	10.44

**Table 8:** Comparison of signal-reconstruction and diagnostic performance for noisy, wavelet-denoised, and Wave-U-Net-denoised signals.

SNR (dB)	Noisy SI-SDR	Wavelet SI-SDR	Wave-U-Net SI-SDR	Noisy Acc.	Wavelet Acc.	Wave-U-Net Acc.
0	−0.004	−1.462	8.807	69.266	48.403	97.371
−2	−2.006	−3.425	7.222	62.738	43.770	96.518
−4	−4.007	−5.429	5.482	57.688	40.069	94.901
−6	−6.010	−7.469	3.549	52.669	37.927	90.337
−8	−8.013	−9.538	1.445	47.728	36.151	79.812
−10	−10.017	−11.633	−0.726	41.280	34.385	65.159

**Table 9:** Classification performance recovery under valve and pump noise at an input SNR of 0 dB.

Classifier	Input	Noise Type			
		Valve Noise		Pump Noise	
		Noisy (%)	Denoised (%)	Noisy (%)	Denoised (%)
CNN	Time	50.36	96.93	38.75	95.06
	STFT	88.63	95.71	80.83	94.35
LSTM	Time	48.33	97.08	46.13	96.55
	STFT	93.87	97.26	85.30	96.55
CNN-LSTM	Time	46.37	95.54	37.50	93.33
	STFT	93.63	96.25	85.38	93.69
Average		70.20	96.46	62.39	94.92

**Table 10:** Grad-CAM cosine similarity to the noiseless reference for Time-CNN and STFT-CNN at an input SNR of 0 dB.

Model	Class	Noisy	Denoised	*n*
Time-CNN	Normal	0.832 ± 0.092	0.966 ± 0.037	420
	Electrical	0.540 ± 0.186	0.967 ± 0.038	414
	Bearing	0.515 ± 0.195	0.962 ± 0.042	406
	Eccentricity	0.479 ± 0.183	0.837 ± 0.132	409
	Overall	0.593 ± 0.220	0.933 ± 0.092	1649
STFT-CNN	Normal	0.964 ± 0.109	0.991 ± 0.040	365
	Electrical	0.991 ± 0.052	0.996 ± 0.032	410
	Bearing	0.997 ± 0.025	0.999 ± 0.005	418
	Eccentricity	0.982 ± 0.067	0.992 ± 0.035	413
	Overall	0.984 ± 0.070	0.995 ± 0.031	1606

**Table 11:** Silhouette scores of the high-dimensional feature spaces for the six classification models under noiseless, noisy, and denoised conditions at 0 dB.

Domain	Model	Silhouette Score		
		Noiseless	Noisy	Denoised
Time	CNN	0.620	0.064	0.589
	LSTM	0.852	−0.067	0.841
	CNN-LSTM	0.891	0.022	0.859
Frequency	CNN	0.708	0.561	0.659
	LSTM	0.897	0.736	0.866
	CNN-LSTM	0.907	0.723	0.843

## Data Availability

The data presented in this study are available from the corresponding author upon reasonable request.
